# Both IRBIT and long-IRBIT bind to and coordinately regulate Cl^−^/HCO_3_^−^ exchanger AE2 activity through modulating the lysosomal degradation of AE2

**DOI:** 10.1038/s41598-021-85499-6

**Published:** 2021-03-16

**Authors:** Ryo Itoh, Naoya Hatano, Momoko Murakami, Kosuke Mitsumori, Satoko Kawasaki, Tomoka Wakagi, Yoshino Kanzaki, Hiroyuki Kojima, Katsuhiro Kawaai, Katsuhiko Mikoshiba, Koichi Hamada, Akihiro Mizutani

**Affiliations:** 1 Department of Pharmacotherapeutics, Showa Pharmaceutical University, Machida, Tokyo 194-8543 Japan; 2 Division of Applied Cell Biology, Graduate School of Interdisciplinary Science and Engineering in Health Systems, Okayama University, Okayama, 700-8530 Japan; 3 Laboratory of Cell and Tissue Biology, Keio University School of Medicine, Tokyo, 160-8582 Japan; 4 Shanghai Institute for Advanced Immunochemical Studies, ShanghaiTech University, Shanghai, 201210 China

**Keywords:** Ion channel signalling, Stress signalling

## Abstract

Anion exchanger 2 (AE2) plays crucial roles in regulating cell volume homeostasis and cell migration. We found that both IRBIT and Long-IRBIT (L-IRBIT) interact with anion exchanger 2 (AE2). The interaction occurred between the conserved AHCY-homologous domain of IRBIT/L-IRBIT and the N-terminal cytoplasmic region of AE2. Interestingly, AE2 activity was reduced in L-IRBIT KO cells, but not in IRBIT KO cells. Moreover, AE2 activity was slightly increased in IRBIT/L-IRBIT double KO cells. These changes in AE2 activity resulted from changes in the AE2 expression level of each mutant cell, and affected the regulatory volume increase and cell migration. The activity and expression level of AE2 in IRBIT/L-IRBIT double KO cells were downregulated if IRBIT, but not L-IRBIT, was expressed again in the cells, and the downregulation was cancelled by the co-expression of L-IRBIT. The mRNA levels of AE2 in each KO cell did not change, and the downregulation of AE2 in L-IRBIT KO cells was inhibited by bafilomycin A1. These results indicate that IRBIT binding facilitates the lysosomal degradation of AE2, which is inhibited by coexisting L-IRBIT, suggesting a novel regulatory mode of AE2 activity through the binding of two homologous proteins with opposing functions.

## Introduction

IRBIT (IP_3_R binding protein released with inositol 1,4,5-trisphosphate) was identified as a molecule that regulates Ca^2+^ concentration by competing with IP_3_ for the IP_3_ receptor^1,2^ and is now considered to be a multifunctional protein because of its wide range of target molecules^3^. IRBIT is a phosphoprotein and its phosphorylation is required for the binding to some target proteins^2,4^, and IRBIT itself interacts with some protein/lipid kinases and phosphatases and is involved in the modulation of phosphorylation signals^3,5–8^. Considering that the activities of diverse ion transporters are regulated by IRBIT in a phosphorylation-dependent manner, IRBIT can function both as a sensor and an integrating modulator of the intracellular ionic milieu^9,10^.

Long-IRBIT (L-IRBIT) is a homolog of IRBIT, and both share a highly conserved C-terminal S-adenosylhomocysteine hydrolase (AHCY) domain and N-terminally adjacent multiple-phosphorylation sites, but the latter has a distinct longer N-terminal stretch^11^. Previous reports showed that L-IRBIT has a different binding affinity or functional effect on the target molecules of IRBIT^11–13^. Both IRBIT and L-IRBIT show a ubiquitous tissue expression, but the expression level of IRBIT is generally much higher than that of L-IRBIT. At the cellular level, however, L-IRBIT shows a more restricted expression pattern than IRBIT, for example, the specific expression in some interneurons of the cerebellum^11^. A recent report of splicing variants of L-IRBIT with different N-terminal sequences has shown that each variant has unique properties in protein stability, target molecule spectrum, and functional consequence^14^. Together, IRBIT and L-IRBIT can form homo- or hetero-multimers via conserved AHCY domains. The “IRBIT family” is supposed to serve as a hub protein and to be involved in diverse cellular functions, especially in the regulation of the intracellular ionic milieu^7,14^.

Intracellular ion homeostasis is fundamental to maintain not only proper biochemical reactions, but also a normal cell morphology and behavior. In particular, cell volume regulation is a dynamic process that requires the actions of many ion channels and transporters^15^. For example, in regulatory volume increase (RVI), which is triggered by exposing cells to hypertonic environments, stimulation of Na^+^/K^+^/2Cl^−^ cotransporters (mainly NKCC1), or a parallel activation of Na^+^/H^+^ exchangers (NHEs mainly, NHE1) and Cl^−^/HCO_3_^−^ anion exchangers (AEs mainly, AE2) is involved^15,16^. Both NHEs and AEs are also major regulators of the intracellular pH, generally acting as acid extruders and acid loaders, respectively^17^, and these ion transporters contribute to maintaining global intracellular ion and pH homeostasis. Furthermore, a polarized expression of these transporters in a cell allows them to function locally and helps cell migration by generating local cell volume changes and/or intracellular pH gradients. IRBIT family proteins bind to and regulate some of these ion transporters, being involved in cell volume regulation or cell migration; however, no direct evidence of their involvement has yet been reported.

In this study, to investigate the participation of IRBIT family proteins in such cellular functions, we attempted to identify the binding proteins of the IRBIT family in B16-F10 murine melanoma cells, which are known for their highly metastatic nature^18^. We found that both IRBIT and L-IRBIT bound to SLC4A2 gene product, anion exchanger 2 (AE2), through their conserved AHCY domain. We generated IRBIT-, L-IRBIT-, or IRBIT/L-IRBIT double-knockout B16–F10 cells, and examined AE2 activity and its associated cell function in each mutant cell. Interestingly, AE2 activity was reduced only in L-IRBIT KO cells, and RVI and cell migration of L-IRBIT KO cells were also impaired. The reduction of AE2 activity in L-IRBIT KO cells was due to the decrease in AE2 protein expression level, which was restored by treatment with bafilomycin A1, a specific inhibitor of lysosome H^+^-ATPase. The exogenous expression of IRBIT or/and L-IRBIT in IRBIT/L-IRBIT double-knockout cells clearly showed that the downregulation of AE2 was facilitated by IRBIT homomultimer, which was inhibited by the co-expression of L-IRBIT. These results imply a novel mode of regulation of AE2 activity, which was achieved by IRBIT family, based on the modulation of the stability of its proteins.

## Results

### AE2 is a novel target molecule of IRBIT family proteins

**Figure 1:**
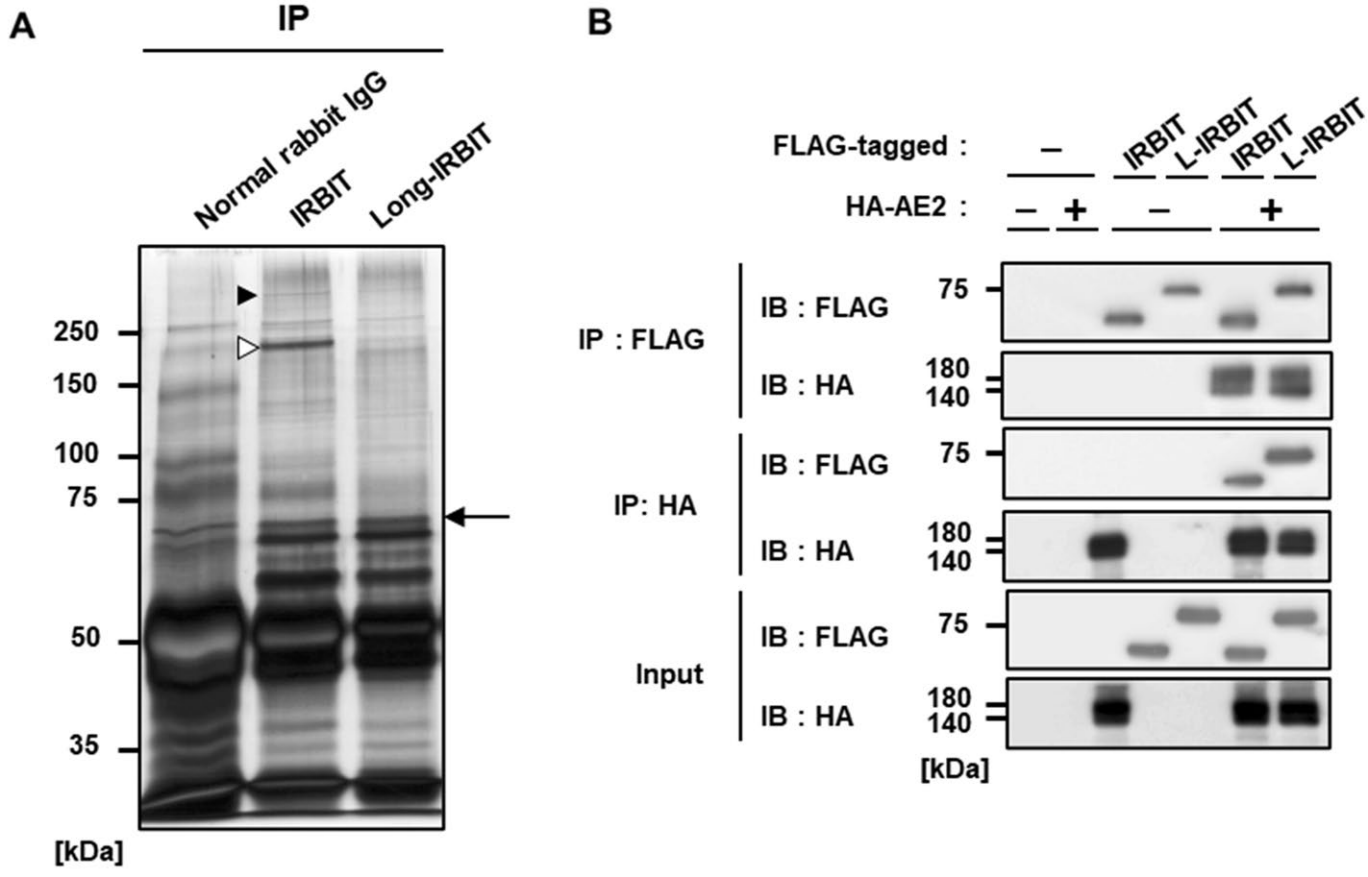
IRBIT family proteins bind to AE2. (**A**) Proteins bound to IRBIT and L-IRBIT in B16-BL6 cells obtained by the immunoprecipitation using anti-IRBIT and anti-L-IRBIT antibodies, respectively, were visualized using silver staining. Protein bands indicated by arrowheads and an arrow were processed for LC–MS/MS analysis. (**B**) FLAG-tagged IRBIT or FLAG-tagged L-IRBIT was transfected into HEK 293 T cells alone or together with HA-tagged AE2. Each lysate expressing each construct (input) was processed for immunoprecipitation with the indicated antibody (IP). Co-immunoprecipitates were analyzed using immunoblot with the indicated antibodies (IB).

We attempted to find IRBIT family binding proteins in B16-F10 cells using a co-immunoprecipitation assay with anti-IRBIT and L-IRBIT antibodies. Several proteins were found to be specifically co-precipitated with IRBIT or L-IRBIT (Fig. 1A). Each band indicated by an arrow or arrowheads on the SDS-PAGE was analyzed using LC–MS/MS analysis, and the identified proteins are listed in Table 1. Among them, AE2, which was identified from the IRBIT coprecipitate (Fig. 1A, closed arrowhead), is well known for its involvement in cell migration and cell volume regulation, and we focused on its interaction with the IRBIT family.

**Table 1:** IRBIT family binding protein in B16F10 mouse melanoma cell line.

No.	Swiss prot ID	Protein name	Molecular weights	Mascot score
(1)	B3A2_MOUSE	Anion exchange protein 2	136,728	24
(2)	A2M_MOUSE	Alpha-2-macroglobulin	165,748	41
	LAMC2_MOUSE	Laminin subunit gamma-2	130,077	16
(3)	GRP75_MOUSE	Stress-70 protein, mitochondrial	73,416	315
	SAHH3_MOUSE	Putative adenosylhomocysteinase 3	66,857	137
	HSP7C_MOUSE	Heat shock cognate 71 kDa protein	70,827	47
	SGG1_MOUSE	Secretogranin-1	77,922	19
	LMNA_MOUSE	Prelamin-A/C	74,193	18

Each number indicates an arrow or arrowhead in Fig. 1A (1): closed arrowhead (2): open arrowhead (3): closed arrow.

First, to confirm the binding between AE2 and IRBIT family proteins, a co-immunoprecipitation assay using HEK 293 T cells overexpressing HA-tagged AE2 and FLAG-tagged IRBIT family proteins was carried out. AE2 was co-precipitated with either IRBIT or L-IRBIT and, conversely, IRBIT and L-IRBIT were co-precipitated with AE2 to the same extent (Fig. 1B), indicating that AE2 is a binding target molecule of both IRBIT and L-IRBIT.

### AE2 activity is reduced in L-IRBIT knockout cells

**Figure 2:**
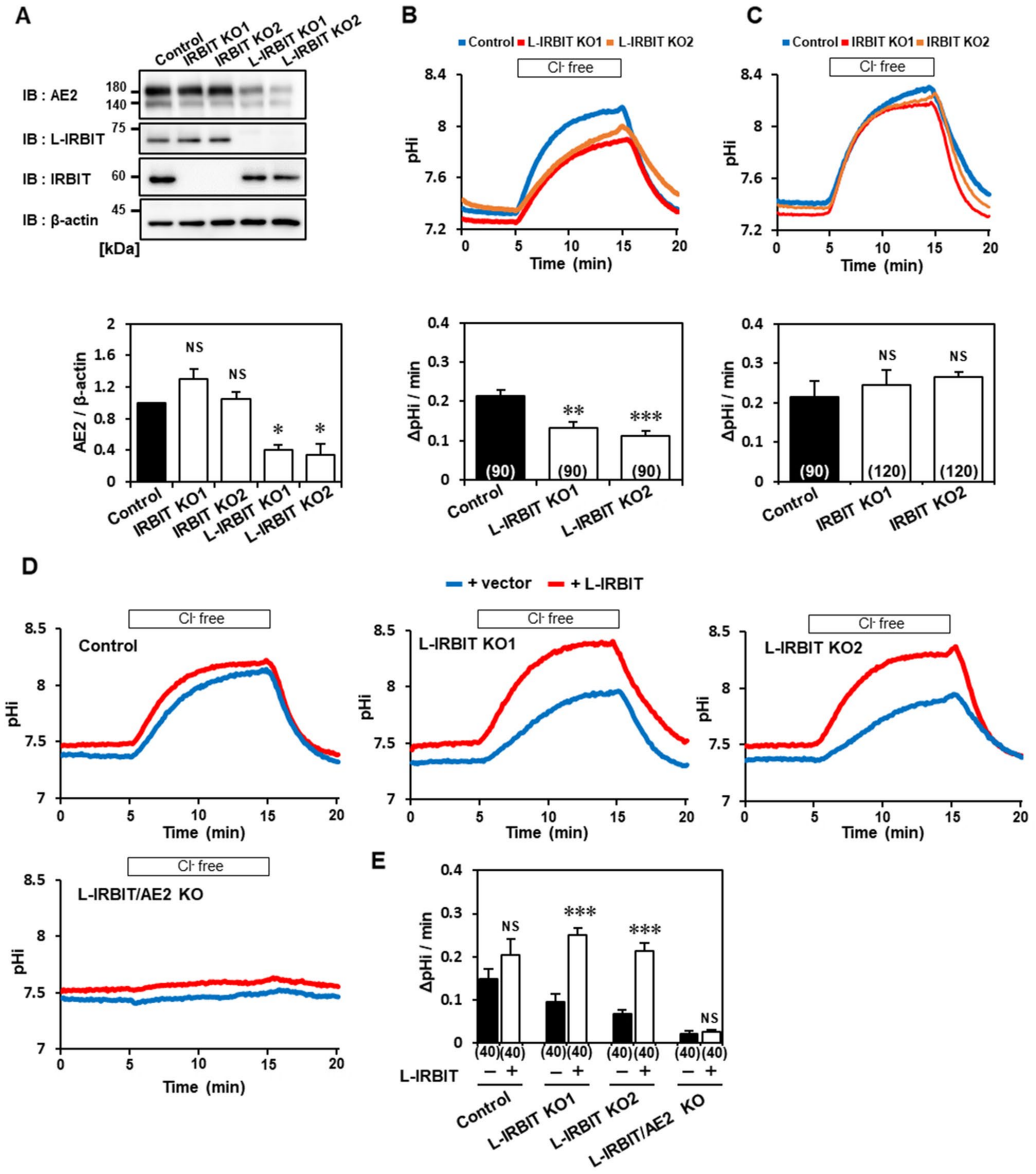
AE2 activity is downregulated in L-IRBIT knockout cells, and the activity is rescued by the exogenous expression of L-IRBIT. (**A**) IRBIT- or L-IRBIT knockout (KO) cells were established using CRISPR/Cas9 strategy and two independent clones of each KO cell line were verified for the expressions of IRBIT, L-IRBIT, and AE2 using an immunoblot (upper panel). Relative expression level of AE2 in each KO cells compared with that of control cells is shown, N = 4 (lower panel). (**B**) AE2 activity in L-IRBIT KO cells was examined by measuring the intracellular pH change (ΔpHi) upon changing the perfusion buffer from Cl^–^ containing to Cl^–^ free ringer buffer containing SNARF1 pH sensitive dye. Representative plots of pHi change obtained from control (blue), L-IRBIT KO1 (red), and L-IRBIT KO2 (orange) (upper panel). The average AE2 activity (∆pHi/min) of each cell type was 0.21 ± 0.02 (WT), 0.13 ± 0.01 (L-IRBIT KO1), and 0.11 ± 0.01 (L-IRBIT KO2), N = 3 (lower panel). (**C**) A representative plot of pHi change obtained from control (blue), IRBIT KO1 (red), and IRBIT KO2 (orange) (upper panel). The AE2 activity was 0.22 ± 0.04 (WT), 0.25 ± 0.04 (IRBIT KO1), or 0.27 ± 0.01 (IRBIT KO2), respectively, N = 4 (lower panel). (**D**) The effects of the exogenous expression of L-IRBIT on AE2 activity in each cell type, WT, L-IRBIT KO1, L-IRBIT KO2, or L-IRBIT/AE2 double knockout (DKO). L-IRBIT expressing cells were selected based on the co-expressed GFP signals. Blue trace is the mock control and the red trace is L-IRBIT expressing WT B16–F10 cells (upper-left panel), L-IRBIT KO1 cells (upper-middle panel), L-IRBIT KO2 cells (upper-right panel), and L-IRBIT/AE2 double knockout cells (lower-left panel). (**E**) Average AE2 activity of each cell type was 0.15 ± 0.02 (WT + vector), 0.20 ± 0.04 (WT + L-IRBIT), 0.10 ± 0.02 (L-IRBIT KO1 + vector), 0.25 ± 0.02 (L-IRBIT KO1 + L-IRBIT), 0.07 ± 0.01 (L-IRBIT KO2 + vector), 0.21 ± 0.02 (L-IRBIT KO2 + L-IRBIT), 0.02 ± 0.01 (L-IRBIT/AE2 DKO + vector), 0.03 ± 0.01(L-IRBIT/AE2 DKO + L-IRBIT), N = 4. The total cell numbers were indicated in each graph. *P < 0.05, **P < 0.01, ***P < 0.001, NS (no significance).

To understand the effects of IRBIT or L-IRBIT on AE2 function, we established IRBIT family knockout cells using the CRISPR/Cas9 system. We confirmed the absence of IRBIT or L-IRBIT in two independently established clones. Surprisingly, the level of AE2 expression changed in an opposite manner in each knockout cell. That is, AE2 expression was slightly increased in IRBIT knockout cells; on the other hand, it was significantly decreased in L-IRBIT knockout cells (Fig. 2A). Next, we measured AE2 activity in each knockout cell. AE2 activity was represented by intracellular alkalization upon changing the perfusing solution from a Cl^–^containing one to a Cl^–^ free one. In control (wild type) cells, a robust alkalinization was observed using this protocol and the alkalinization was abolished in AE2 knockout cells (Fig. S1A–C), indicating that the alkalinization in B16-F10 cells was fully derived from AE2. AE2 activity in L-IRBIT knockout cells was significantly decreased (Fig. 2B). We noticed that both clones of IRBIT KO showed slight increase in baseline pHi and AE2 activity, however the difference was not reaching statistical significance (Fig. 2C).

To confirm the effects of L-IRBIT KO on AE2 activity, we examined whether AE2 activity was rescued when L-IRBIT was exogenously expressed in L-IRBIT KO cells. If L-IRBIT was exogenously expressed in each L-IRBIT KO cell clone, AE2 activity was significantly increased in both L-IRBIT KO clones. Furthermore, when L-IRBIT was overexpressed in control cells, AE2 activity was slightly increased, and neither the overexpression nor the knockout of L-IRBIT led to changes in AE2 KO cells (Figs. 2D and S1A–C). These results clearly indicated that the loss of L-IRBIT in B16–F10 cells decreased AE2 expression, resulting in the reduction of AE2 activity.

### AE2-associated cellular processes are impaired in L-IRBIT knockout cells

**Figure 3:**
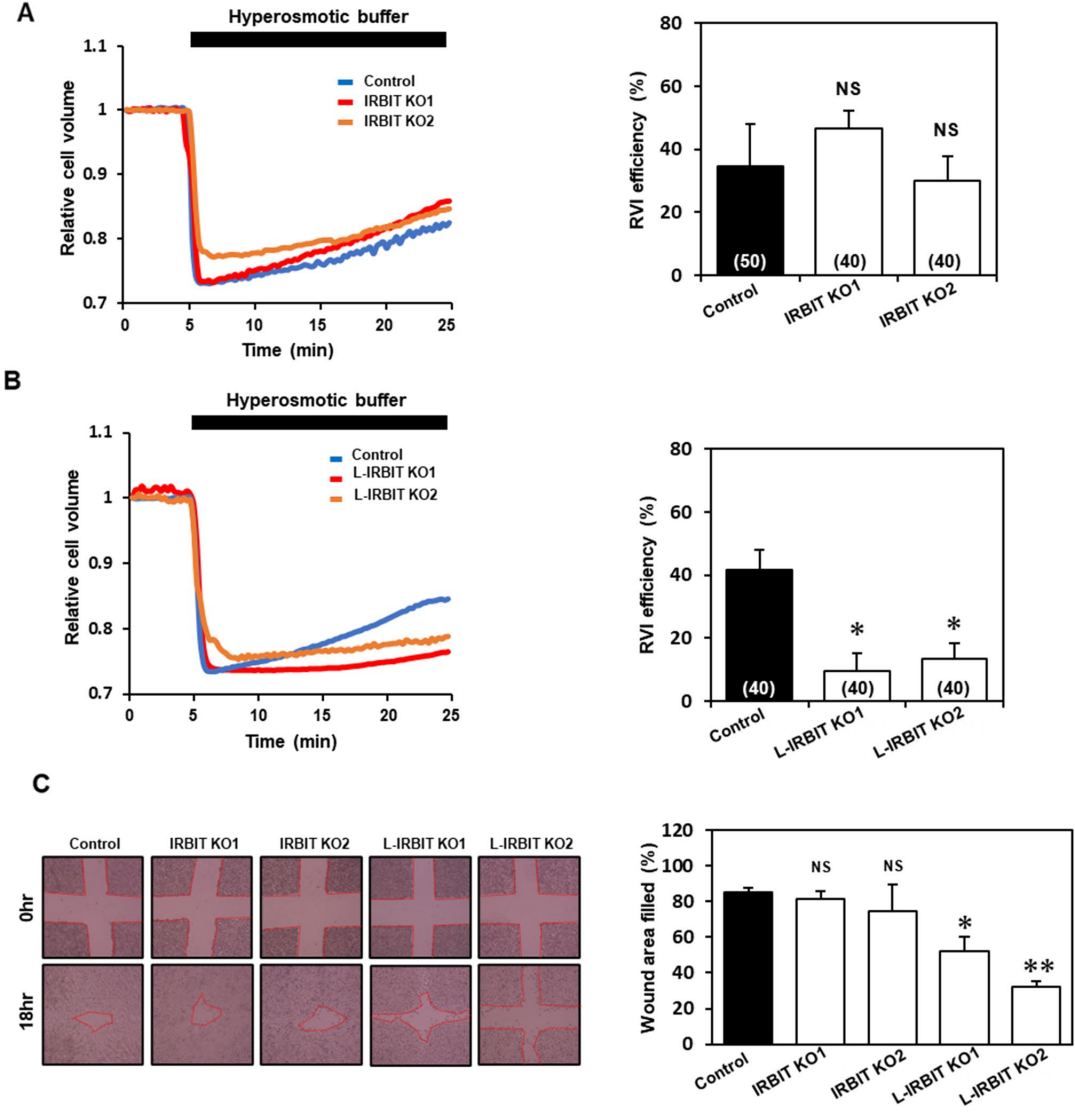
Cell volume recovery after hypertonic stress is impaired in L-IRBIT knockout cells. (**A**) Cell volume recovery in IRBIT KO cells was measured based on the fluorescence change upon changing the perfusion buffer from a 300 mOsm buffer to a 450 mOsm buffer using calcein-AM. Representative plots of the relative cell volume change compared with a baseline obtained from the control (blue), IRBIT KO1 (red), and IRBIT KO2 (orange) (left panel). RVI efficiency of each cell type at 20 min after replacing the buffer with a 450 mOsm buffer. RVI efficiency (%) was 34.6 ± 13.6 (WT), 46.7 ± 5.6 (IRBIT KO1), 30.0 ± 7.7 (IRBIT KO2), N = 3–4 (right panel). (**B**) Cell volume recovery in L-IRBIT KO cells was measured as above. A representative plot of the relative cell volume from control (blue), L-IRBIT KO1 (red), and L-IRBIT KO2 (orange) (left panel). RVI efficiency of each cell type was 41.8 ± 6.1 (WT), 9.7 ± 5.6 (L-IRBIT KO1), 13.3 ± 5.0 (L-IRBIT KO2). N = 3—4 (right panel). (**C**) The results of the wound healing assay were shown. Representative photomicrographs of the wounded cell monolayer are shown (left panel). Wound width was measured in 6 positions immediately after wounding and 18 h later in WT, IRBIT KO (IRBIT KO1, IRBIT KO2) and L-IRBIT KO (L-IRBIT KO1, L-IRBIT KO2), N = 4 (right panel). The total cell numbers were indicated in each graph. *P < 0.05, **P < 0.01, NS (no significance).

AE2 is involved in cell volume recovery after hyperosmotic stimulation by taking up Cl^-^ into cells^19^. To evaluate the contribution of L-IRBIT to cell volume regulation by regulating AE2 activity, we examined the cell volume recovery after hyperosmotic stimulation using the calcein quenching method^20^. Cell volume recovery was observed after changing the osmolarity of perfusion buffers from 300 to 450 mOsm in control cells; however, it was almost abolished in AE2 KO cells (Fig. S1D), indicating that the cell volume recovery observed in these experimental conditions was attributed to AE2 activity (Fig. S1E). In IRBIT knockout cells, the recovery rate was not significantly different from that of control cells (Fig. 3A). On the other hand, L-IRBIT knockout cells showed a significantly reduced cell volume recovery (Fig. 3B). These results suggest that L-IRBIT may be involved in cell volume regulation through the modulation of AE2 activity.

AE2 is localized at the leading edge of migrating fibroblasts, and its activity is also required for cell migration^21,22^. Thus, we also examined the migration of each KO cell using a scratch assay. The assay was carried out under reduced-serum (0.5%) medium conditions, at which no significant difference in proliferation speed was observed among the KO cells (Fig. S2A and B). AE2 KO cells showed a reduced migration compared to control cells, indicating that AE2 is involved in cell migration of these melanoma cells (Fig. S2C,D). Cell migration of L-IRBIT KO cells (L-IRBIT KO1 and L-IRBIT KO2) was significantly reduced (Fig. 3C), and double knockout of AE2 and L-IRBIT did not show any effects on cell migration, suggesting that the effect of the deletion of L-IRBIT on cell migration was mediated by AE2. In contrast, IRBIT KO cells showed a similar cell migration rate compared to control cells (Fig. 3C).

Taken together, L-IRBIT KO cells showed a decrease in AE2 expression and a reduction in its associated cellular functions, implying a specific potential role of L-IRBIT in modulating the activity of AE2.

### L-IRBIT binds to AE2 through direct interaction of the common AHCY domain of IRBIT family and the N-terminal region of AE2

**Figure 4:**
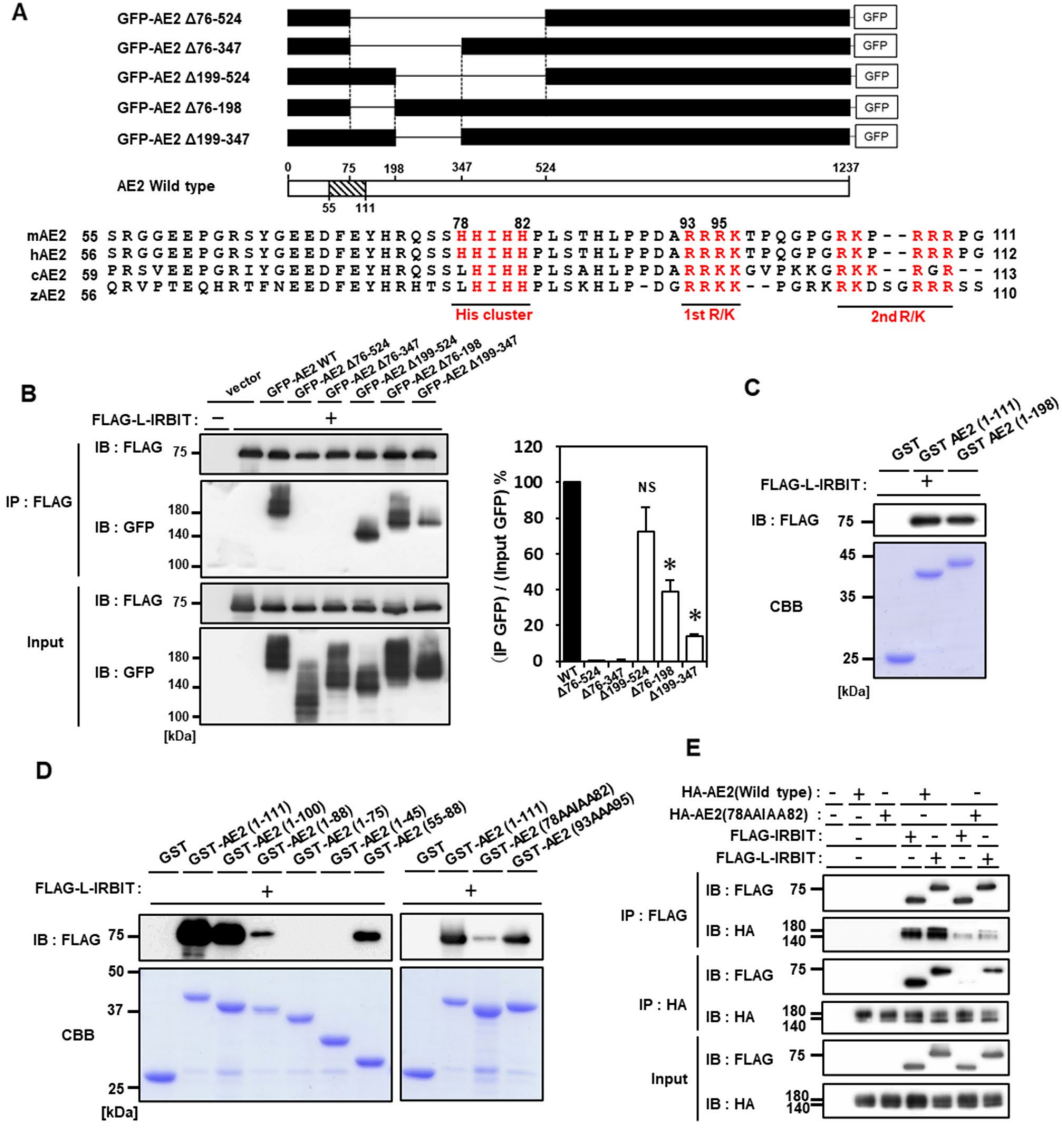
His cluster in the N-terminal region of AE2 is involved in the interaction between AE2 and IRBIT family proteins. (**A**) Schematic diagram of GFP-tagged AE2 deletion mutants (upper panel). Sequence alignment of the N-terminal regions (aa 55–111, in mouse) of various species AE2. Red letters indicate clusters of basic amino acids and are designated as His cluster, 1st R/K cluster, and 2nd R/K cluster, respectively. mAE2: mouse AE2, NM_009207.3; hAE2: humanAE2, NM_003040.4; cAE2: chickenAE2, NM_204963.1; zAE2: zebrafish AE2, NM_001037237.1 (Lower panel). (**B**) FLAG-tagged L-IRBT was transfected into HEK 293 T cells alone or with GFP-tagged AE2. The lysate expressing each construct (input) was processed for immunoprecipitation with each indicated antibody (IP). Co-immunoprecipitation was analyzed using immunoblots with the indicated antibodies (IB) (left panel). Binding efficiency of each truncated AE2 mutant to L-IRBIT was calculated based on the GFP signals of co-immunoprecipitated/those of input. Relative binding efficiency of each truncated AE2 mutant is represented by the percentage of that of wild type AE2 (100%), N = 3. *P < 0.05, NS (no significance) (right panel). (**C**, **D**) FLAG-tagged L-IRBIT was transfected into HEK 293 T cells, and the lysate was pulled down by each GST-tagged fusion protein carrying the indicated N-terminal region with or without AE2 mutations. Bound L-IRBIT was examined using immunoblotting and anti-FLAG antibody. (**E**) HA-tagged AE2 wild type or mutant (aa 78–82) was transfected into HEK 293 T cells alone or with FLAG-tagged IRBIT family. The lysate expressing each construct (input) was processed for immunoprecipitation with each indicated antibody (IP). Co-immunoprecipitation was analyzed using immunoblots with the indicated antibodies (IB).

It is well known that IRBIT binds to and activates NBCe1B, a gene product of SLC4A4. In this case, IRBIT directly binds to the splicing-specific N-terminal cytoplasmic region of NBCe1B^4^. AE2 is also classified in the SLC4A gene family (being also referred to as SLC4A2), and it has similar structural features to those of NBCe1B^23,24^. Thus, we predicted that the region to which L-IRBIT binds would be within the long cytoplasmic N-terminal region of AE2, and we performed a co-immunoprecipitation binding assay using a series of N-terminal deletion mutants of AE2 (Fig. 4A). As shown in Fig. 4B, the deletion of an N-terminal region ranging from aa 76–524, L-IRBIT no longer bound to the deleted form of AE2. L-IRBIT did not bind to AE2 mutants lacking the aa 76–347 sequence either. The AE2 mutant lacking aa 199–524 sequence bound to L-IRBIT to a similar extent as wild-type AE2, suggesting that the binding site was within aa 76–199. However, the AE2 mutant lacking aa 76–198 sequence still bound to L-IRBIT, but this binding strength was lower than that of AE2 lacking aa 199–524, suggesting there would be two separate L-IRBIT binding sites in the N-terminus of AE2, namely aa 76–199 and aa 199–347 (Fig. 4A). Indeed, if aa 199–347 is deleted from AE2, the mutant shows a much weaker binding strength to L-IRBIT than wild-type AE2. This implies that aa 348–524 might behave as an inhibitory region for L-IRBIT binding to aa 76–199, and that the aa 199–347 region might also have a role as a suppressor of the aa 348–524-mediated inhibition.

NBCe1B has characteristic clusters of positively charged residues in the N-terminal region^25^. It has been reported that IRBIT binds to and enhances NBCe1B and that the three conserved arginine residues in the N-terminal region are essential for the interaction between IRBIT and NBCe1B^25^. In analogy with these findings, we examined the N-terminal sequence of AE2 and found clusters of basic residues in mouse AE2, which are conserved among various species (Fig. 4A). Thus, we focused on the N-terminal region from aa 1–198, and prepared GST-fusion proteins carrying various parts of the N-terminal aa 1–198 of AE2 and examined the interaction between GST fusion proteins and full-length FLAG-tagged L-IRBIT expressed in HEK 293 T cells using a GST pull-down assay. As expected, GST-tagged AE2-aa1-198 bound to L-IRBIT (Fig. 4C). GST-tagged AE2-aa1-111 bound to L-IRBIT to a similar extent as GST-tagged AE2-aa1-198, suggesting that aa 112–198 is not involved in binding (Fig. 4C). AE2-aa1-111 indeed includes clusters of basic residues, which are separated into three parts: His cluster, 1st Arg cluster, and 2nd Arg cluster (Fig. 4A). To investigate which part(s) is (are) critical for the L-IRBIT binding, fusion proteins of GST-tagged AE2 were further truncated and examined for the binding to L-IRBIT. A pull-down assay demonstrated the robust binding of GST-tagged AE2-aa1-100 to L-IRBIT, suggesting that the 2nd Arg cluster was not involved in the interaction, while GST-tagged AE2-aa1-88 showed a reduced binding (Fig. 4D). This suggested that the 1st Arg cluster was significantly involved in the interaction. If deleted further, GST fusion proteins, either GST-tagged AE2-aa1-75 or GST-tagged AE2-aa1-45, no longer bound to L-IRBIT, indicating that the His cluster is indispensable for the interaction (Fig. 4D). The critical involvement of His cluster in the interaction was supported by the finding that GST-tagged AE2-aa55-88, which only includes His cluster and its N-terminal flanking region, showed a substantial binding to L-IRBIT (Fig. 4D). We further examined the importance of the His cluster and the 1st Arg cluster for the interaction with point mutations in basic residues. In the pull-down assay, both mutants of GST-tagged AE2-aa 1–111, GST-tagged AE2-aa 78AAIAA82 and GST-tagged AE2-aa 93AAA95, showed a significantly reduced binding to L-IRBIT, and the reduction was much more obvious in GST-tagged AE2-aa 78AAIAA82 than in GST-tagged AE2-aa 93AAA95, suggesting that the His cluster had a greater contribution to the interaction (Fig. 4D). Indeed, heterologous co-expression and immunoprecipitation experiments showed that the binding of full-length AE2 mutated in the aa 78–82 region to both IRBIT and L-IRBIT was much weaker compared to that of wild-type AE2 (Fig. 4E). These results indicated that, for the interaction between AE2 and the IRBIT family proteins, basic amino acids in the N-terminal region of AE2 are important for the binding to NBCe1B; however, His residues of AE2 were more critical than Arg clusters.

**Figure 5:**
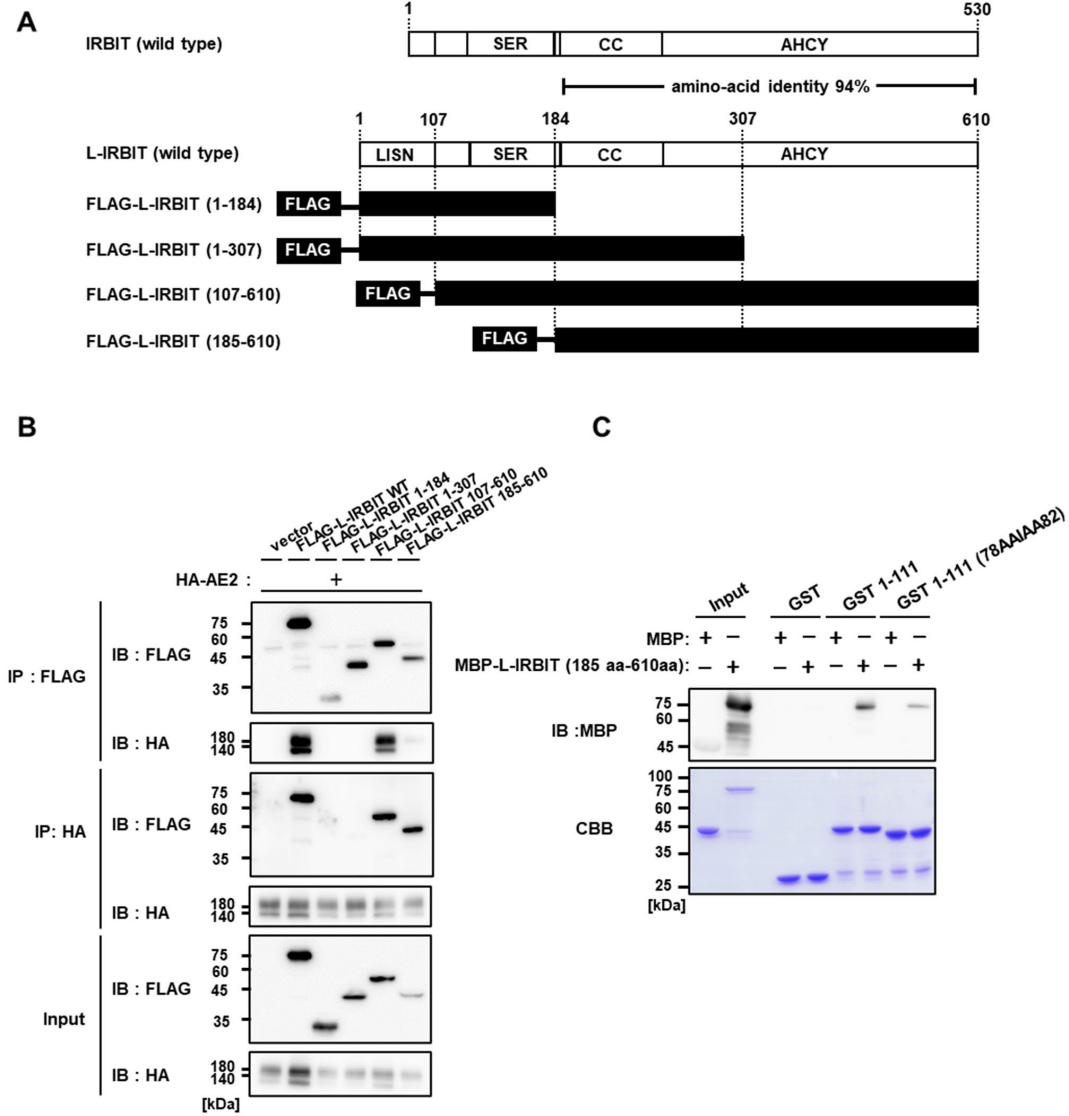
The C-terminal AHCY domain of IRBIT family proteins directly interacts with the N-terminal region of AE2. (**A**) Schematic structure of IRBIT family proteins and the truncated mutants of FLAG-tagged L-IRBIT. LISN: Long-IRBIT specific N-terminal domain; SER: serine-rich region; CC: coiled-coil region; AHCY domain: adenosyl homocysteine hydrolase-like domain are indicated. (**B**) HA tagged AE2 wild type was transfected into HEK 293 T cells alone or with FLAG-tagged L-IRBIT wild type or with FLAG-tagged L-IRBIT deletion mutants (aa 1–184, aa 1–307, aa 107–610, and aa 185–610). The lysate expressing each construct (input) was processed for immunoprecipitation with each indicated antibodies (IP). Co-immunoprecipitates were analyzed using immunoblots with the indicated antibodies (IB). (**C**) Purified MBP fusion protein (MBP or MBP-tagged L-IRBIT) was pulled down by using purified GST fusion protein (GST, GST-tagged-AE2 (aa 1–111), or GST-tagged-AE2 (aa 78–82). Bound proteins were examined using immunoblotting with anti-MBP antibody.

Next, to determine the region of IRBIT family proteins responsible for AE2 binding, a series of deletion mutants of FLAG-tagged L-IRBIT were prepared (Fig. 5A) and were checked for their binding to full-length AE2 in a heterologous expression context. The binding between HA-tagged AE2 and full-length FLAG-tagged L-IRBIT was confirmed using directional co-immunoprecipitation assays. If deletion occurred in the IRBIT-family conserved coiled-coil plus the AHCY domain (FLAG-tagged L-IRBIT-aa1-184 in Fig. 5B) or the C-terminal part of AHCY domain (FLAG-tagged L-IRBIT-aa1-307), the binding between HA-tagged AE2 and truncated FLAG-tagged L-IRBIT was no longer observed. In contrast, the LISN region, L-IRBIT specific appendage part, was dispensable for the binding (FLAG-tagged L-IRBIT-aa107-610), which is consistent with the finding that both IRBIT and L-IRBIT have a comparable binding capability to AE2, as indicated above. A further N-terminally truncated form of FLAG-tagged L-IRBIT [FLAG-tagged L-IRBIT (aa 185–610)], lacking conserved multiple phosphorylation sites required for the binding to various target proteins, such as IP_3_R and NBCe1C^2,4^, still bound to AE2. These results suggest that, for the binding of IRBIT family proteins to AE2, the conserved C-terminal region, including the coiled-coil region and the AHCY domain of the IRBIT family proteins is essential, and that the phosphorylation of IRBIT family proteins is not necessary.

To confirm the direct and phosphorylation-independent binding of the IRBIT family proteins to AE2, we prepared purified proteins carrying each essential part from *E. coli* and examined the binding using a pull-down experiment. Control maltose binding protein (MBP) was not pulled down by either GST or GST-tagged AE2-aa1-111. However, the MBP fusion protein carrying L-IRBIT-aa185-610, which showed no binding to GST, was pulled down by GST-tagged AE2-aa1-111 (Fig. 5C). GST-tagged AE2-aa1-111 mutated in Hiss to Alas (78AAIAA82) showed a reduced binding to MBP-tagged L-IRBIT-aa185-610. These results clearly demonstrate that the IRBIT family directly binds to AE2 through the interaction between the conserved C-terminal domain of the IRBIT family proteins and the N-terminal His cluster region of AE2.

### IRBIT homomultimer facilitates the lysosomal degradation of AE2, which is suppressed by the incorporation of L-IRBIT into the multimer

**Figure 6:**
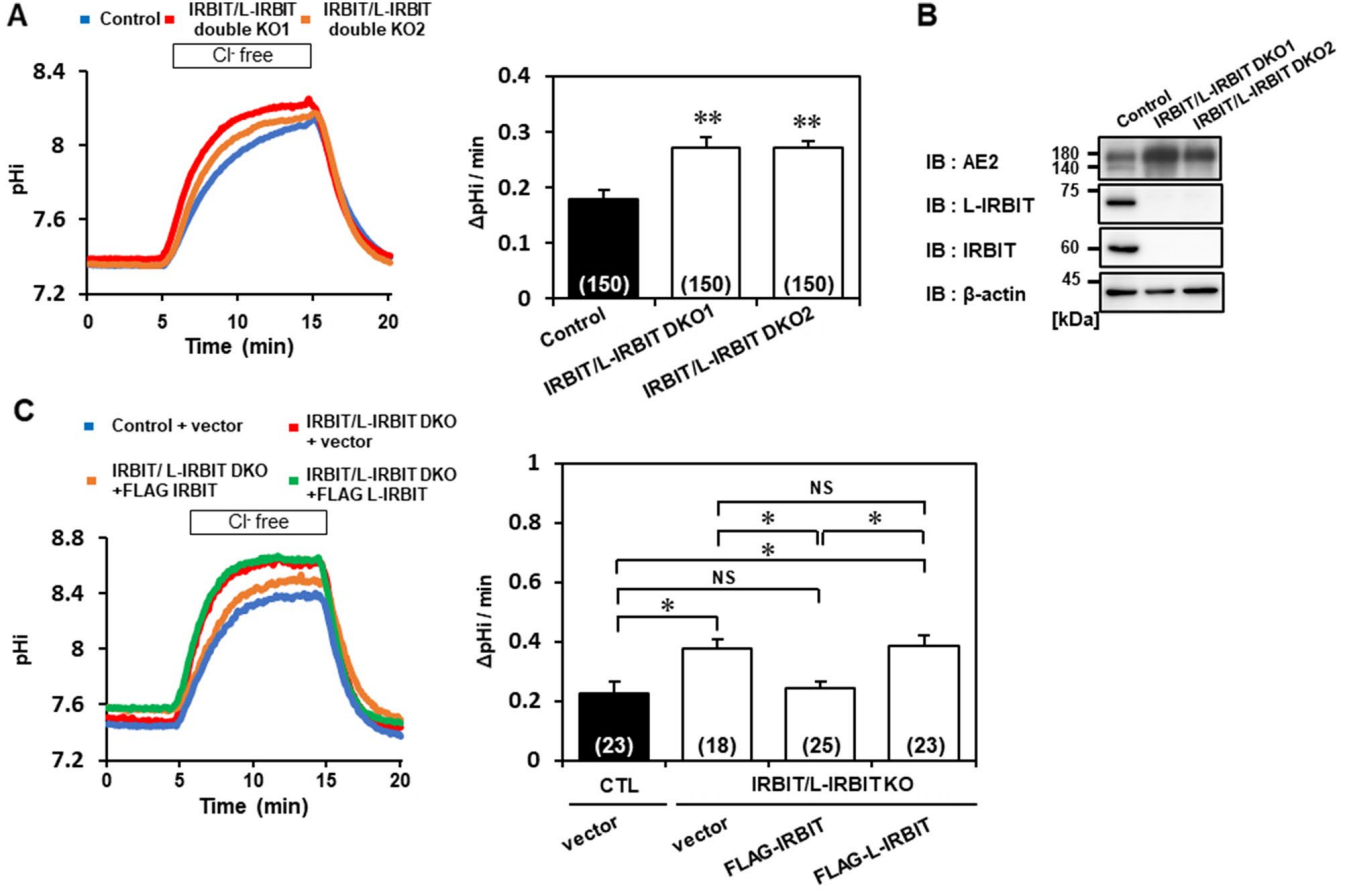
IRBIT homomultimer decreases the stability and activity of AE2. (**A**) IRBIT/L-IRBIT double KO cells were established using CRISPR/Cas9 strategy, and AE2 activity was measured in the cells. A representative plot of pHi change obtained from control (blue), IRBIT/L-IRBIT DKO clone 1 (red), and IRBIT/L-IRBIT DKO clone 2 (orange) (left panel). The average AE2 activity (ΔpH/min) was 0.17 ± 0.01 (WT), 0.26 ± 0.02 (IRBIT/L-IRBIT DKO1), and 0.24 ± 0.01 (IRBIT/L-IRBIT DKO2), N = 5 (right panel). (**B**) Protein expression of IRBIT, L-IRBIT, and AE2 in IRBIT/L-IRBIT double DKO cells was verified using immunoblotting. (**C**) The effects of the exogenous expression of IRBIT or L-IRBIT on AE2 activity in IRBIT/L-IRBIT DKO cells were analyzed. IRBIT or L-IRBIT expressing cells were selected based on the GFP co-expressed signals. Blue trace is the control cells, a red trace is the IRBIT/L-IRBIT DKO cells, an orange trace is IRBIT/L-IRBIT DKO cells expressed with IRBIT, and a green trace is IRBIT/L-IRBIT DKO cells expressed with L-IRBIT (left panel). Average AE2 activity in each cell type was 0.22 ± 0.04 (WT + vector), 0.37 ± 0.03 (IRBIT/L-IRBIT DKO + vector), 0.24 ± 0.02 (IRBIT/L-IRBIT DKO + IRBIT), 0.35 ± 0.02 (IRBIT/L-IRBIT DKO + L-IRBIT), N = 4—5 (right panel). The total cell numbers were indicated in each graph. **P < 0.01, NS (no significance).

As shown above, AE2 is a common binding target of the IRBIT family. However, the reduction in the expression level and the consequent decrease in the transporter activity of AE2 was found only in L-IRBIT knockout cells, but not in IRBIT knockout cells. To clarify the mechanism that explains the discrepancy between the binding capacity and functional properties of IRBIT and L-IRBIT to AE2, we established IRBIT/L-IRBIT double knockout cells and measured AE2 activity using SNARF-1. Interestingly, AE2 activity in IRBIT/L-IRBIT double knockout cells was increased by 1.4-fold compared to control cells (Fig. 6A). Consistent with this, the expression level of AE2 in IRBIT/L-IRBIT double knockout cells was increased in parallel (Fig. 6B), and the migration of IRBIT/L-IRBIT double knockout cells was comparable to that of control cells (Fig. S2E–G). Together, the results of IRBIT family KO cells suggested that IRBIT can function as a negative regulator of AE2 expression level and that L-IRBIT serves as an endogenous competitor for IRBIT. Thus, we tried to express IRBIT or L-IRBIT in double KO cells and examined AE2 activity. As expected, if IRBIT was expressed in double KO cells, AE2 activity was reduced (Fig. 6C). Meanwhile, if L-IRBIT was expressed, AE2 activity did not change (Fig. 6C). Interestingly, both IRBIT- and L-IRBIT-expressed cells showed increase in baseline pHi. Although the mechanism was not clear, it might be related to the fact that IRBIT family have multiple target ion transporters and show different actions towards them^14^.

**Figure 7:**
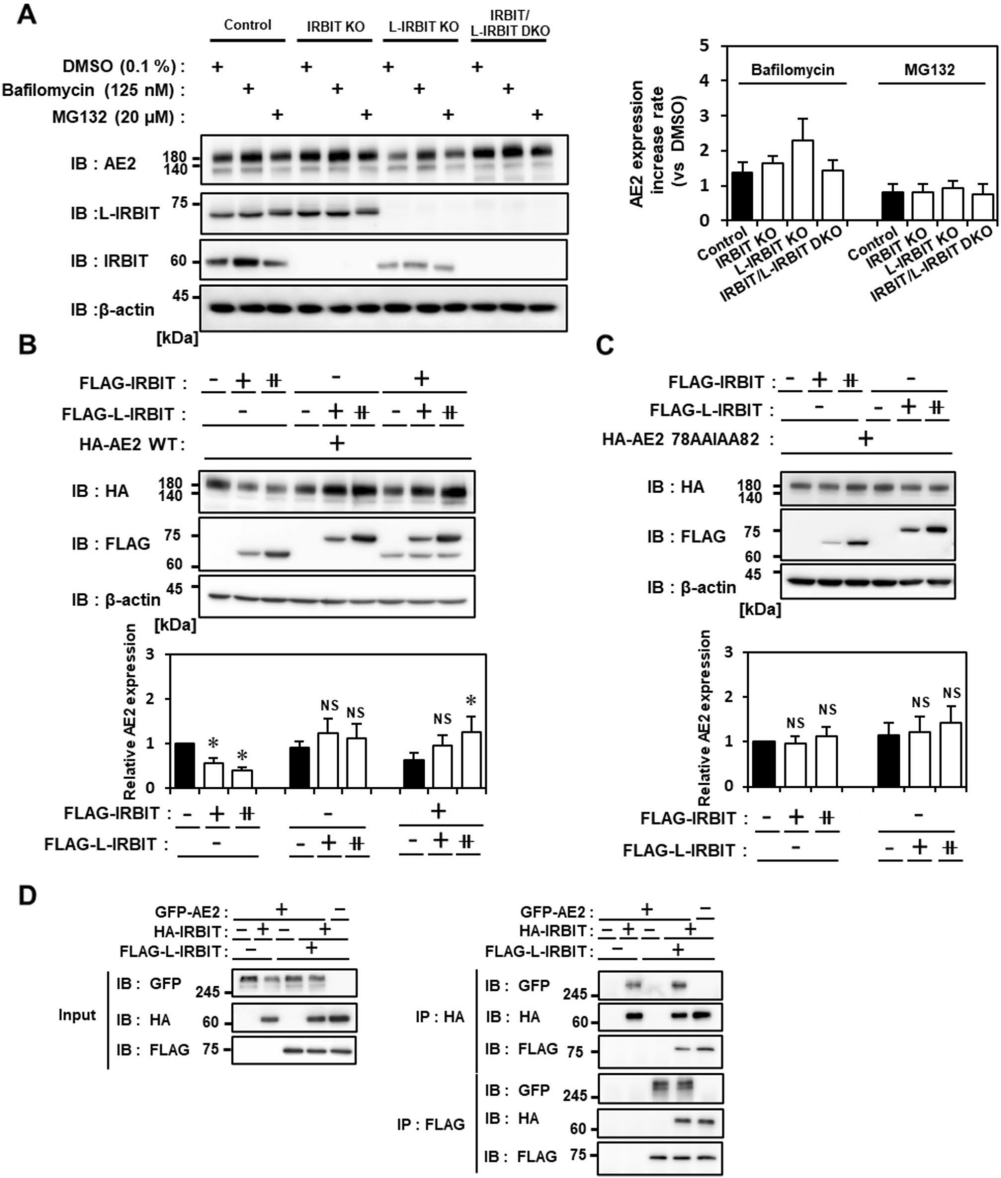
IRBIT homomultimer binding facilitates the degradation of AE2 through lysosomal degradation pathway, and the incorporation of L-IRBIT into the multimer suppresses AE2 degradation. (**A**) Each of IRBIT family knockout cells were treated with DMSO, Bafilomycin A1 (125 nM) or MG132 (20 μM) for 3 h (left panel). The effects of compounds on the expression level of AE2 in each KO cells were evaluated by comparing AE2 expression in compound-treated cells to that in DMSO control cells, which is represented as fold increase, N = 5 (right panel). (**B**) The expression level of AE2 exogenously expressed in IRBIT/L-IRBIT DKO cells was examined when IRBIT and/or L-IRBIT were co-expressed with a different combination ratio. Expression levels of AE2, IRBIT, and L-IRBIT were evaluated using an immunoblot with anti-HA antibody (AE2) and anti-FLAG antibody (IRBIT and L-IRBIT). “−”, “+”, “⧺” indicate the amount of each plasmid DNA used in transfection. Relative expression level of HA-tagged AE2 is shown, N = 3 (lower panel). (**C**) Expression level of mutant AE2 (aa 78–82) in IRBIT/L-IRBIT DKO cells co-expressed with FLAG-tagged IRBIT or L-IRBIT was examined. Relative expression level of mutant HA-tagged AE2 are shown, N = 3 (lower panel). (**D**) Binding of multimers of IRBIT family proteins to AE2 were examined. GFP-tagged AE2 wild type was transfected into IRBIT/L-IRBIT DKO cells with HA-tagged IRBIT and/or with FLAG-tagged L-IRBIT. The lysate expressing each construct (input) was processed for immunoprecipitation with each anti-HA antibody or anti-FLAG antibody (IP). Co-immunoprecipitates were analyzed using an immunoblot with the indicated antibodies (IB). *P < 0.05, NS (no significance).

It is well known that the IRBIT family proteins form a multimer via the C-terminal AHCY domain^11^. Indeed, our co-immunoprecipitation analysis demonstrated that IRBIT and L-IRBIT existed as hetero-multimers (Fig. 1A and Table 1). AE2 expression level and activity in L-IRBIT KO cells, but not in double KO cells, was significantly decreased, suggesting that IRBIT homo-multimer may downregulate AE2 expression. To clarify whether this downregulation occurs at the transcription level, we examined AE2 mRNA levels in each KO cell using real-time PCR. The AE2 mRNA level was almost constant in each knockout cell (Fig. S3A,B). This suggests that IRBIT homo-multimers may influence the stability of AE2 protein. Thus, we then examined the effects of the chemical compounds on AE2 protein level using bafilomycin A1 (125 nM) and MG132 (20 μM), which are inhibitors of lysosomal degradation or proteasomal degradation, respectively. As shown in Fig. 7A, AE2 protein level was increased by 1.5-fold in bafilomycin A1-treated control cells, but not in MG132-treated control cells. In L-IRBIT-KO cells, if treated with bafilomycin A1, AE2 protein levels were increased by 2.2-fold and were restored to the control level. In contrast, MG132 treatment did not show any changes in the AE2 protein levels in L-IRBIT-KO cells, suggesting that IRBIT homo-multimer binding to AE2 facilitates AE2 degradation via the endocytosis/lysosome pathway. MG132 treatment in IRBIT/L-IRBIT double KO and IRBIT KO cells showed no increase in the AE2 protein levels; however, bafilomycin treatment increased AE2 protein levels again in these cells. This increase was to a lower extent than in L-IRBIT KO cells, suggesting that AE2 may be continuously down-regulated via IRBIT-independent and endocytosis/lysosome-dependent degradation pathway.

To directly examine the effects of IRBIT homomultimers on AE2 protein stability, AE2 and IRBIT were exogenously expressed in IRBIT/L-IRBIT double knockout cells with a stepwise increase in IRBIT expression. The expression level of AE2 decreased, as that of IRBIT increased (Fig. 7B). On the other hand, the expression level of AE2 did not change or rather increased if L-IRBIT was exogenously expressed (Fig. 7B). In addition, the decrease in AE2 expression induced by IRBIT expression was rescued by the co-expression of L-IRBIT, indicating that IRBIT homomultimer binding facilitated the degradation of AE2, which was inhibited by the co-existence of L-IRBIT (Fig. 7B). The AE2 mutant (aa 78–82) showing a reduced binding to IRBIT was not affected in its expression level if it was co-expressed with IRBIT or L-IRBIT in IRBIT/L-IRBIT double knockout cells, suggesting that the degradation of AE2 was indeed induced by IRBIT binding (Fig. 7C). Contrasting to the finding that full-length L-IRBIT expression in L-IRBIT KO cells rescued the AE2 activity (Fig. 2D), expression of mutant IRBIT family proteins lacking AE2 binding, IRBIT (aa 1–227) or L-IRBIT (aa 1–307), did not induce any change of AE2 activity (Fig. S4A,B), suggesting that the effects of IRBIT family on the stability and activity of AE2 was mediated by the direct binding. Moreover, If IRBIT or L-IRBIT was immunoprecipitated from the IRBIT/L-IRBIT double knockout cells exogenously expressed with AE2, IRBIT and L-IRBIT were demonstrated to form hetero-multimers, which bound to AE2 to a similar extent as IRBIT homomultimers or L-IRBIT homo-multimers (Fig. 7D), suggesting that the incorporation of L-IRBIT into multimers diminished the degradative action of IRBIT homomultimers.

All these data suggest that the binding of IRBIT homo-multimer facilitates endocytosis/lysosome-dependent degradation of AE2 and that L-IRBIT plays a role as a dominant negative regulator of IRBIT action.

## Discussion

We have identified AE2 as a novel target molecule of IRBIT family proteins. IRBIT and L-IRBIT show a similar binding ability to AE2. Interestingly, we found that IRBIT homo-multimer binding facilitates the degradation of AE2 via the endocytosis/lysosome pathway, thereby dampening the AE2 activity and its associated cellular function. Furthermore, the incorporation of L-IRBIT into the multimer inhibited AE2 degradation, indicating that IRBIT and L-IRBIT have opposing effects regarding AE2 stability. Given that the expression ratio of IRBIT/L-IRBIT differs in a cell-type-dependent manner and can change according to the cellular context, both IRBIT and L-IRBIT may coordinately contribute to cell type- and/or cellular context-specific regulation of AE2 activity.

In this study, we identified AE2 as a common binding target for both IRBIT and L-IRBIT, which was supported by the results of biochemical binding experiments, demonstrating that the conserved AHCY domain of IRBIT/L-IRBIT was responsible for the binding to AE2. As AHCY forms a multimer^26,27^, IRBIT/L-IRBIT also forms homo- or hetero-multimers with each other through the AHCY domain, and AE2 binds to every multimer, namely IRBIT homomultimer, L-IRBIT homomultimer, or IRBIT/L-IRBIT hetero-multimer, with similar binding affinities (Fig. 7). Among them, only IRBIT homomultimer binding facilitated the degradation of AE2 through the endocytosis/lysosome pathway, whose mechanism is still not fully understood. However, considering that the target molecules of IRBIT reported previously, such as IP_3_R and NBCe1-B, did not show any evidence of degradation upon interaction with IRBIT^2,4,28^, the endocytosis/lysosomal degradation of AE2 induced by IRBIT homomultimer binding is likely attributed to some specific properties of AE2 complexed with IRBIT homomultimer and/or to some unknown characteristics of B16–F10 cells, which were used in this study. These issues should be elucidated in future experiments.

To our knowledge, this type of regulation for ion-transporters by two homologous binding proteins with opposing functions may be a first case. In other fields of biology, there is a similar example, a case of ASPP (apoptosis stimulating protein of p53) and iASPP (inhibitory member of the ASPP family). ASPP and iASPP are homologous proteins, both of which bind to p53. Through their binding to p53, ASPP promotes apoptosis, while iASPP suppress apoptosis^29–31^. Our findings with ASPP/iASPP case might imply potential evolutionary significance of gene duplication and alternative splicing.

Recently, we reported splice variants of L-IRBIT having a shorter N-terminal region than that of IRBIT, L-IRBIT V3, and L-IRBIT V4^14^. These variants can mimic the IRBIT function and influence AE2 activity. However, given that authentic L-IRBIT (L-IRBIT V1 or L-IRBIT V2) was predominantly expressed in B16–F10 cells, and that L-IRBIT KO cells were produced using a guide RNA sequence that targets a common exon of all L-IRBIT variants, our results likely reflect the effects of IRBIT and authentic L-IRBIT. However, as shown in a previous report^14^, short variants of L-IRBIT show a more marked cell type-specific expression. Thus, especially in some cell types, the contribution of short-form L-IRBIT variants to AE2 regulation should be verified in future studies.

IRBIT homodimer-mediated degradation of AE2 was induced by IRBIT binding to the N-terminal cytoplasmic region of AE2. We found that at least two regions, namely aa 76–199 and aa 199–347, were IRBIT binding sites on AE2. In this study, we focused on the N-terminal binding site, aa 76–199, which includes three tandem clusters of basic amino acids and we found that the His cluster was a critical site for IRBIT binding. Considering that these clusters of basic amino acids are conserved among the species (Fig. 4A), the regulation of AE2 expression and activity by the IRBIT family would likely be conserved beyond species. Several cases of IRBIT binding to basic amino acid clusters on its target molecules have been documented, and most of them are dependent on the phosphorylation of IRBIT; for example, IRBIT binding to IP_3_R or to NBCe1^2,4^. In the case of the binding to AE2, phosphorylation of IRBIT/L-IRBIT seemed to be not required. However, examining the effects of the phosphorylation of IRBIT/L-IRBIT on the binding affinity to AE2 and/or the efficiency of AE2 degradation would be intriguing to seek a more dynamic regulation of AE2.

In RVI, parallel activation of NHEs and AEs is involved. Hyperosmotic stress activates NHEs and H^+^ is extruded out of cells, resulting in the increase of pHi. The increase of pHi is balanced by AE2 activation leading to efflux of HCO_3_^−^ and accumulation of Cl^-^. In this study, AE2 KO cells did not show complete abolishment of RVI (Fig. S1D and E), probably due to compensatory mechanisms of other ion transporters, such as activation of NKCC and NHEs activation. Considering that IRBIT family proteins, especially the L-IRBIT V3 which is a potent activator of NHE3, also bind to and modulate the activity of NHE3^14,32^, The effects of L-IRBIT KO on RVI might also include the change of NHE activity induced by L-IRBIT KO.

It has been reported that the N-terminal cytoplasmic region of AE2 is responsible for its activation upon intra- and extracellular changes in pH or osmolarity. The core responsible region is identified to be the aa 300–400 region of AE2^19,23,33–35^. This N-terminal region partially overlaps with the secondary IRBIT binding site, aa 199–347, which we did not address in this study. Considering that changes in the extracellular pH or osmolarity induce the activation of multiple signaling pathways, including phosphorylation/dephosphorylation events, it would be worthwhile to test the phosphorylation dependency of the interaction between IRBIT and the secondary binding site of AE2, aa 190–347 and the possibility of involvement of IRBIT in the activation of AE2 upon pH or osmolarity changes.

Many types of ion transporters play a crucial role in cell migration processes, under both physiological and pathophysiological conditions, and AE2 is one of them^36,37^. Indeed, we confirmed the importance of AE2 in cell migration, as shown by the phenotype of AE2 knockout B16-F10 cells (Fig. S2C,D), and several pieces of evidence indicated that IRBIT family proteins participate in the regulation of cell migration by modulating AE2 expression level. We addressed the overall cellular expression levels of AE2; however, for directional cell migration, an asymmetrical localization of AE2 and its localized activity are crucial^21,22^. Furthermore, AE2 KO mice display abnormalities such as achlorhydria^38^, osteopetrosis^39^ and fertility associated with testicular dysplasia ^40^, suggesting the possible involvement of IRBIT family proteins in the regulatory mechanism of acid secretion, osteoclast differentiation, and spermatogenesis. Thus, in future research, we intend to clarify the effects of IRBIT family proteins on the subcellular expression of AE2 and the involvement of this regulation in pathophysiological conditions, which may lead to the discovery of potential therapeutic targets for cancer metastasis and the elucidation of its pathophysiology.

## Materials and methods

### Cell culture and transfection

B16-F10 melanoma cell lines were kindly provided by Dr. Susumu Itoh (Showa Pharmaceutical University, Tokyo, Japan). B16–F10 and HEK-293 T cells were maintained in Dulbecco’s modified Eagle’s medium (DMEM) supplemented with 10% fetal bovine serum, 50 units/mL penicillin, and 50 μg/mL streptomycin. B16–F10 and HEK-293 T cells were transiently transfected with various constructs using polyethylenimine (Polysciences).

### Plasmids, recombinant proteins

Mammalian expression plasmids encoding FLAG-tagged IRBIT wild type and FLAG-tagged L-IRBIT wild type were described previously^2,11^. FLAG-tagged L-IRBIT deletion mutants (aa 1–184, aa 1–307, aa 107–610, and aa 185–610) were constructed using PCR-based site-directed mutagenesis. The full-length murine AE2 cDNA was purchased from Danaform. cDNA encoding N-terminally HA-tagged AE2 was amplified using PCR and subcloned into the pcDNA3.1 (+) vector (Life Technologies).

Bacterial expression plasmids encoding various N-terminal parts of AE2 and various regions of L-IRBIT were constructed by subcloning each fragment amplified using PCR into *E. coli* expression vector pGEX5x-3 (GE Healthcare) and pMAL-c5x (New England BioLabs), respectively. The DNA sequences of all constructed plasmids were verified using DNA sequencing.

### Antibodies

The antibodies used were rabbit anti-IRBIT antibody^1^ and rabbit anti-L-IRBIT antibody^11^. Rabbit anti-AE2 (Santa Cruz, cat.# sc-376632), mouse anti-FLAG antibody (cat.# 014-22383), rabbit anti-HA antibody (Roche, cat.# 11867423001), mouse anti-GFP antibody (Santa Cruz, cat.# sc-9996), mouse anti-β-actin antibody (Santa Cruz, cat.# sc-69879), mouse anti-MBP antibody (Santa Cruz, cat.# sc-13564) were purchased.

### Generation of IRBIT, L-IRBIT, or AE2 knockout B16-F10 cells using CRISPR/Cas9-mediated genome editing

The target sequences for CRISPR interference were designed using the CRISPR direct^41^. The target sequences used for mouse IRBIT, L-IRBIT, and AE2 were CCCTACTAAGACTGGCCGGAGAT (IRBIT KO1, IRBIT KO2), TGGCAAGAGGATAGTACTGCTGG (L-IRBIT KO1), LCAGAGCAGATTCCGTTAGGCAGG (L-IRBIT KO2), and CCTCCAGGAGGCTGGATCCCGGG (AE2). Two complementary oligonucleotides with BpiI restriction sites for guide RNAs (gRNAs) were synthesized at Sigma-Aldrich, and cloned into the pX459 CRISPR/Cas9-Puro vector (Addgene).

### Immunoblotting

Proteins were separated using SDS-PAGE and transferred to a polyvinylidene difluoride membrane. The membrane was blocked with 5.0% (wt/vol) skim milk in PBS for 1 h and probed with the primary antibody overnight at 4 °C. After washing with PBS containing 0.05% (wt/vol) Tween-20 (PBST), the membranes were incubated with an appropriate HRP-conjugated secondary antibody, and the signals were detected using Immoblilon Forte Western HRP substrate (Millipore).

### Immunoprecipitation

For immunoprecipitation, HEK 293 T cells expressing FLAG-tagged L-IRBIT and HA-tagged AE2 were washed with PBS and solubilized in lysis buffer (10 mM HEPES [pH 7.4], 100 mM NaCl, 2 mM EDTA, 0.1% Triton X-100, 10 mM sodium fluoride, protease inhibitor cocktail (EDTA free) (Nacalai Tesque). The homogenate was centrifuged at 20,000×*g* for 15 min. The supernatant was precleared with Protein G Sepharose (GE Healthcare) and incubated with the appropriate antibodies and Protein-G overnight at 4 °C. The beads were then washed five times with lysis buffer, and the proteins were eluted by boiling in SDS/PAGE sampling buffer.

### Intracellular pH imaging

Intracellular pH was measured by recording 5-(and-6)-carboxy SNARF-1-AM (Roche) fluorescence intensities at emission wavelengths of 580 and 640 nm with an excitation wavelength of 488 nm using a confocal microscope (A1R, Nikon Instruments). These cells were perfused with Ringer’s buffer containing the following: 5 mM glucose, 5 mM potassium gluconate, 1 mM calcium gluconate, 1 mM MgSO_4_, 2.5 mM NaH_2_PO_4_・2H_2_O, 25 mM NaHCO_3_, 10 mM HEPES (pH 7.4, NaOH), containing 140 mM NaCl (Cl^–^ containing), or 140 mM sodium gluconate (Cl^–^ free). For intracellular pH imaging, cells were grown on poly L-lysine-coated glass 24 h before. SNARF-1 (20 μM) was loaded via Cl^-^containing Ringer’s buffer and incubated for 10 min at room temperature. During the measurement, the perfusion solutions were continuously bubbled with 5% CO_2_ at 37 °C. For stabilization, the cells were perfused with Cl^-^ containing Ringer’s buffer for 10 min, and the intracellular pH was measured at 6 s intervals. After perfusing the cells with Cl^−^ containing Ringer’s buffer for 5 min, the buffer was replaced with Cl^–^ free Ringer’s buffer for 10 min. For quantification, regions of interest (ROIs) were placed on individual cells, and fluorescence signals were extracted from each ROI. In one experiment, 30 cells were analyzed. The mean values of pHi from 3 independent experiments were plotted. AE2 activity was determined via linear regression of alkalinization from each cells^42^. pHi was represented by the SNARF-1 fluorescence ratio (640 nm/580 nm)^43^, and calibration was performed with 11 μM nigericin in HEPES buffer: 126 mM NaCl, 4.4 mM KCl, 11 mM glucose, 1.1 mM CaCl_2_, 1 mM MgCl_2_, and 24 mM HEPES-Na at pH 6.8, 7.0, 7.4, 7.8, and 8.0.

### Cell volume measurement

Cell volume changes in B16-F10 cells were measured using the calcein self-quenching method^20^. These cells were perfused with isotonic buffer or hypertonic buffer containing the following: 10 mM glucose, 2 mM KCl, 2 mM KH_2_PO_4_, 2 mM CaCl_2_, 2 mM MgCl_2_, 20 mM D-mannitol, 25 mM NaHCO_3_, and 10 mM HEPES (pH 7.4, NaOH), containing either 130 mM NaCl (isotonic) or 205 mM NaCl (hypertonic). For cell volume measurement under hypertonic stress, cells were seeded on poly L-lysine-coated glass covereslips 24 h before. Prior to measurements, 40 μM calcein-AM (eBioscience) was loaded with isoosmotic buffer and incubated in 5% CO_2_ at 37 °C for 1 h. Before hyperosmotic challenge, the fluorescence (EX: 488 nm, Em: 580 nm, *F*) was measured in 5% CO_2_ at 37 °C for 10 min at 15 s intervals using a confocal fluorescence microscope (A1R, Nikon Instruments). Fluorescence signals were obtained from each ROI, which was placed on individual cells. RVI efficiency (%) shows the recovery rate at 20 min after the cell volume decreases, and it was determined by ((*F*20 min – *F*minimum)/(*F*0 min – *F*minimum)) × 100 using the fluorescence at 0 min (*F*0min), the fluorescence immediately after hypertonic stress (*F*minimum), and the fluorescence at 20 min after hypertonic stress (*F*20min). Mean values of RVI efficiency (%) from 3 independent experiments were plotted. Calibration was performed with 300, 375, 450, 525, and 600 mOsm HEPES buffer: 130 mM, 167.5 mM, 205 mM, 242.5 mM, or 280 mM NaCl, 4.4 mM KCl, 11 mM glucose, 1.1 mM CaCl_2_, 1 mM MgCl_2_, and 24 mM HEPES (pH 7.4, NaOH). *F*/*F*0min was converted to relative cell volume using the value of *F* corresponding to each osmolality (π) and creating a calibration curve (Y axis: *F* (300–600 mOsm)/*F* (300 mOsm), X axis: π (300 mOsm)/π (300–600 mOsm). Using the Y-axis intercept (fb) from the approximate straight line of each plot, the relative cell volume was defined as ((*F*/*F*0) – fb)/(1 – fb).

### Cell proliferation assay

Cells were seeded on 24-well plates at a density of 0.4 × 10^4^ cells per well and incubated at 37 °C with 5% CO_2_ in DMEM with 0.5% FCS. Cells were detached from the flasks using trypsin–EDTA on days 0 and 1, and the cell densities were determined using a hemocytometer.

### Cell migration assay

Cell migration ability was evaluated by scratch migration assay, essentially according to the previous report^35^. That is, B16-F10 cells were seeded to confluence of 4 × 10^5^ cells in 6-well tissue culture plates and the cells were starved overnight in DMEM with 0.5% FCS. A wound was made using a sterile micropipette tip. Floating cells were removed by washing with fresh DMEM supplemented with 0.5% FCS. Cells were photographed immediately after wound initiation, and then at the indicated time points using an inverted CKX41 microscope (Olympus), × 4 objective. Wound healing was quantified using ImageJ software as the percentage of closed wound area.

### GST pull-down assay

GST fusion proteins containing the N-terminal region of AE2 and an MBP fusion protein of L-IRBIT were expressed in *E. coli*. *E. coli* were lysed using ultrasonication in a lysis buffer (40 mM Tris–HCl pH 7.5, 100 mM NaCl, 5 mM EDTA, 0.5% Triton X-100) and the lysates were centrifuged at 10,000×*g* for 15 min. The supernatants were incubated with Glutathione Sepharose 4B beads (GE Healthcare) or amylose resin (New England Biolabs) for 1 h at 4 °C and were washed five times with lysis buffer. The MBP fusion protein bound to amylose was eluted with a lysis buffer containing 10 mM maltose. Transfected HEK 293 T cells were lysed in a lysis buffer (10 mM HEPES [pH 7.4], 100 mM NaCl, 2 mM EDTA, 0.1% Triton X-100, 10 mM sodium fluoride, protease inhibitor cocktail (EDTA free)). Purified MBP fusion protein or HEK 293 T cell lysates were incubated with glutathione Sepharose 4B-binding GST fusion protein for 4 h at 4 °C. After thoroughly washing the glutathione Sepharose 4B, pulled-down proteins were examined using SDS-PAGE, followed by CBB protein staining or immunoblotting.

### Quantitative reverse transcription PCR (qPCR)

Total RNA was isolated from cultured cells using the RNeasy Plus Mini Kit (QIAGEN) according to the manufacturer’s instructions. Total RNA (500 ng) was reverse-transcribed into first-strand complementary DNA (cDNA) using oligo-dT primers and the ReverTra Ace qPCR Master Mix with gDNA Remover (TOYOBO). qPCR was performed using SYBR Green PCR kit (Thermo Fisher Scientific) using StepOne and StepOnePlus Real-Time PCR system (Applied Biosystems). qPCR experiments were performed in triplicates, and the relative expression of RNA was measured using the 2^−ΔΔCt^ method^44^. AE2 mRNA was normalized to glyceraldehyde-3-phosphate dehydrogenase (GAPDH). The specific primers used in the qPCR were as follows: AE2, 5′-GCACCGCAGCTACAACCTTC-3′ and 5′-AGCGTCTGGGCCTCAATCTC-3′^45^; and for GAPDH, 5′-ATCACTGCCACCCAGAAGAC-3′ and 5′-AGGAGACAACCTGGTCCTCA-3′.

### Statistical analysis

All statistical analyses were performed using EZR (Saitama Medical Center, Jichi Medical University, Saitama, Japan)^46^. Significance of the difference between two independent groups of data was analyzed using Student’s t-test. Other statistical data were analyzed using one-way ANOVA and post hoc Dunnett’s multiple comparison or Tukey HSD tests. Each experiment was repeated at least three times, and the data are presented as the mean ± SEM, *P < 0.05, **P < 0.01, and ***P < 0.001.

## Supplementary Information

Supplementary Information.

## Data Availability

All data generated or analyzed during this study are included in this published article and in its supplementary information files.

## References

[1] Ando H, Mizutani A, Matsu-ura T, Mikoshiba K (2003). IRBIT, a novel inositol 1,4,5-trisphosphate (IP_3_) receptor-binding protein, is released from the IP_3_ receptor upon IP_3_ binding to the receptor. J. Biol. Chem..

[2] Ando H (2006). IRBIT suppresses IP_3_ receptor activity by competing with IP_3_ for the common binding site on the IP_3_ receptor. Mol.. Cell.

[3] Kawaai K (2015). IRBIT regulates CaMKIIalpha activity and contributes to catecholamine homeostasis through tyrosine hydroxylase phosphorylation. Proc. Natl. Acad. Sci. USA.

[4] Shirakabe K (2006). IRBIT, an inositol 1,4,5-trisphosphate receptor-binding protein, specifically binds to and activates pancreas-type Na^+^/HCO_3_^−^ cotransporter 1 (pNBC1). Proc. Natl. Acad. Sci. USA.

[5] Devogelaere B (2007). Protein phosphatase-1 is a novel regulator of the interaction between IRBIT and the inositol 1,4,5-trisphosphate receptor. Biochem. J..

[6] Ando H (2015). IRBIT interacts with the catalytic core of phosphatidylinositol phosphate kinase type Ialpha and IIalpha through conserved catalytic aspartate residues. PLoS ONE.

[7] Park S, Hong JH, Ohana E, Muallem S (2012). The WNK/SPAK and IRBIT/PP1 pathways in epithelial fluid and electrolyte transport. Physiology.

[8] Ando H, Kawaai K, Bonneau B, Mikoshiba K (2018). Remodeling of Ca^2+^ signaling in cancer: Regulation of inositol 1,4,5-trisphosphate receptors through oncogenes and tumor suppressors. Adv. Biol. Regul..

[9] Yang D, Shcheynikov N, Muallem S (2011). IRBIT: It is everywhere. Neurochem. Res..

[10] Ando H, Kawaai K, Mikoshiba K (2014). IRBIT: A regulator of ion channels and ion transporters. Biochim. Biophys. Acta.

[11] Ando H, Mizutani A, Mikoshiba K (2009). An IRBIT homologue lacks binding activity to inositol 1,4,5-trisphosphate receptor due to the unique N-terminal appendage. J. Neurochem..

[12] Yamaguchi S, Ishikawa T (2014). AHCYL2 (long-IRBIT) as a potential regulator of the electrogenic Na^+^-HCO_3_^-^ cotransporter NBCe1-B. FEBS Lett..

[13] Park PW, Ahn JY, Yang D (2016). Ahcyl2 upregulates NBCe1-B via multiple serine residues of the PEST domain-mediated association. Korean J. Physiol. Pharmacol..

[14] Kawaai K (2017). Splicing variation of long-IRBIT determines the target selectivity of IRBIT family proteins. Proc. Natl. Acad. Sci. USA.

[15] Hoffmann EK, Lambert IH, Pedersen SF (2009). Physiology of cell volume regulation in vertebrates. Physiol. Rev..

[16] Jentsch TJ (2016). VRACs and other ion channels and transporters in the regulation of cell volume and beyond. Nat. Rev. Mol. Cell Biol..

[17] Casey JR, Grinstein S, Orlowski J (2010). Sensors and regulators of intracellular pH. Nat. Rev. Mol. Cell Biol..

[18] KazukiNakamura NY, Yamaguchi Y, Kagota S, Shnozuka K, Kunitomo M (2002). Characterization of mouse melanoma cell lines by their mortal malignancy using an experimental metastatic model. Life Sci..

[19] Humphreys BD, Jiang L, Chernova MN, Alper SL (1995). Hypertonic activation of AE2 anion exchanger in Xenopus oocytes via NHE-mediated intracellular alkalinization. Am. J. Physiol..

[20] Hamann S (2002). Measurement of cell volume changes by fluorescence self-quenching. J. Fluoresc..

[21] Klein M, Seeger P, Schuricht B, Alper SL, Schwab A (2000). Polarization of Na^+^/H^+^ and Cl^−^/HCO_3_^−^ exchangers in migrating renal epithelial cells. J. Gen. Physiol..

[22] Svastova E (2012). Carbonic anhydrase IX interacts with bicarbonate transporters in lamellipodia and increases cell migration via its catalytic domain. J. Biol. Chem..

[23] Alper SL (2006). Molecular physiology of SLC4 anion exchangers. Exp. Physiol..

[24] Kurtz I, Zhu Q (2013). Structure, function, and regulation of the SLC4 NBCe1 transporter and its role in causing proximal renal tubular acidosis. Curr. Opin. Nephrol. Hypertens..

[25] Hong JH (2013). Convergence of IRBIT, phosphatidylinositol (4,5) bisphosphate, and WNK/SPAK kinases in regulation of the Na^+^-HCO_3_^-^ cotransporters family. Proc. Natl. Acad. Sci. USA.

[26] Mary AT, Borchardt RT, Hershfield MS, DavidSmith G, LynneHowell P (1998). Structure determination of selenomethionyl S-adenosylhomocysteine hydrolase using data at a single wavelength. Nat. Struct. Biol..

[27] Hu Y (1999). Crystal structure of S-adenosylhomocysteine hydrolase from rat liver. Biochemistry.

[28] Yang D (2009). IRBIT coordinates epithelial fluid and HCO_3_^-^ secretion by stimulating the transporters pNBC1 and CFTR in the murine pancreatic duct. J. Clin. Invest..

[29] Bergamaschi D (2003). iASPP oncoprotein is a key inhibitor of p53 conserved from worm to human. Nat. Genet..

[30] Sullivan A, Lu X (2007). ASPP: A new family of oncogenes and tumour suppressor genes. Br. J. Cancer.

[31] Amir AI (2015). Highly homologous proteins exert opposite biological activities by using different interaction interfaces. Sci. Rep..

[32] He P, Klein J, Yun CC (2010). Activation of Na^+^/H^+^ exchanger NHE3 by angiotensin II is mediated by inositol 1,4,5-triphosphate (IP_3_) receptor-binding protein released with IP_3_ (IRBIT) and Ca^2+^/calmodulin-dependent protein kinase II. J. Biol. Chem..

[33] Zhang Y, Chernova MN, Stuart-Tilley AK, Jiang L, Alper SL (1996). The cytoplasmic and transmembrane domains of AE2 both contribute to regulation of anion exchange by pH. J. Biol. Chem..

[34] Stewart AK, Chernova MN, Kunes YZ, Alper SL (2001). Regulation of AE2 anion exchanger by intracellular pH: critical regions of the NH_2_-terminal cytoplasmic domain. Am. J. Physiol. Cell Physiol..

[35] Chernova MN, Jiang AK, Friedman DJ, Kunes YZ, Alper SL (2003). Structure-function relationships of AE2 regulation by Cai^2+^-sensitive stimulators NH^4+^ and hyertonicity. Am. J. Physiol. Cell Physiol..

[36] Webb BA, Chimenti M, Jacobson MP, Barber DL (2011). Dysregulated pH: A perfect storm for cancer progression. Nat. Rev. Cancer.

[37] Morishita K, Watanabe K, Ichijo H (2019). Cell volume regulation in cancer cell migration driven by osmotic water flow. Cancer Sci..

[38] Gawenis LR (2004). Mice with a targeted disruption of the AE2 Cl^−^/HCO_3_^−^ exchanger are achlorhydric. J. Biol. Chem..

[39] Coury F (2013). SLC4A2-mediated Cl^−^/HCO_3_^−^ exchange activity is essential for calpain-dependent regulation of the actin cytoskeleton in osteoclasts. Proc. Natl. Acad. Sci. USA.

[40] Medina JF (2003). Anion exchanger 2 is essential for spermiogenesis in mice. Proc. Natl. Acad. Sci. USA.

[41] Naito Y, Hino K, Bono H, Ui-Tei K (2015). CRISPRdirect: Software for designing CRISPR/Cas guide RNA with reduced off-target sites. Bioinformatics.

[42] Casey JR, Sly WS, Shah GN, Alvarez BV (2009). Bicarbonate homeostasis in excitable tissues: Role of AE3 Cl-/HCO3- exchanger and carbonic anhydrase XIV interaction. Am. J. Physiol. Cell. Physiol..

[43] Hasegawa N (2019). Calcineurin binds to a unique C-terminal region of NBCe1-C, the brain isoform of NBCe1 and enhances its surface expression. BPB Rep..

[44] Livak KJ, Schmittgen TD (2001). Analysis of relative gene expression data using real-time quantitative PCR and the 2^−^^ΔΔCt^ Method. Methods.

[45] Taylor-Burds C, Cheng P, Wray S (2015). Chloride accumulators NKCC1 and AE2 in mouse GnRH neurons: Implications for GABAA mediated excitation. PLoS ONE.

[46] Kanda Y (2013). Investigation of the freely available easy-to-use software 'EZR' for medical statistics. Bone Marrow Transplant.

