# Supplementary material for: Both IRBIT and long-IRBIT bind to and coordinately regulate Cl−/HCO3− exchanger AE2 activity through modulating the lysosomal degradation of AE2
Source: Sci Rep. 2021 Mar 16;11:5990. doi: 10.1038/s41598-021-85499-6 (PMC7966362; doi:10.1038/s41598-021-85499-6)

Supplementary Information

# Both IRBIT and Long-IRBIT bind to and coordinately regulate Cl^-/HCO_3^- exchanger AE2 activity through modulating the lysosomal degradation of AE2

Ryo Itoh[a], Naoya Hatano[b], Momoko Murakami[a], Kosuke Mitsumori[a], Tomoka Wakagi[a], Yoshino Kanzaki[a], Hiroyuki Kojima[a], Katsuhiro Kawaai[c], Katsuhiko Mikoshiba[d], Koichi Hamada[a], and Akihiro Mizutani*[a]

[a]Department of Pharmacotherapeutics, Showa Pharmaceutical University, Machida, Tokyo 194-8543, Japan; [b]Division of Applied Cell Biology, Graduate School of Interdisciplinary Science and Engineering in Health Systems, Okayama University, Okayama 700-8530, Japan; [c]Laboratory of Cell and Tissue Biology, Keio University School of Medicine, Tokyo, 160-8582, Japan; Shanghai Institute for Advanced Immunochemical Studies, ShanghaiTech University, Shanghai, 201210, China

Figure S1

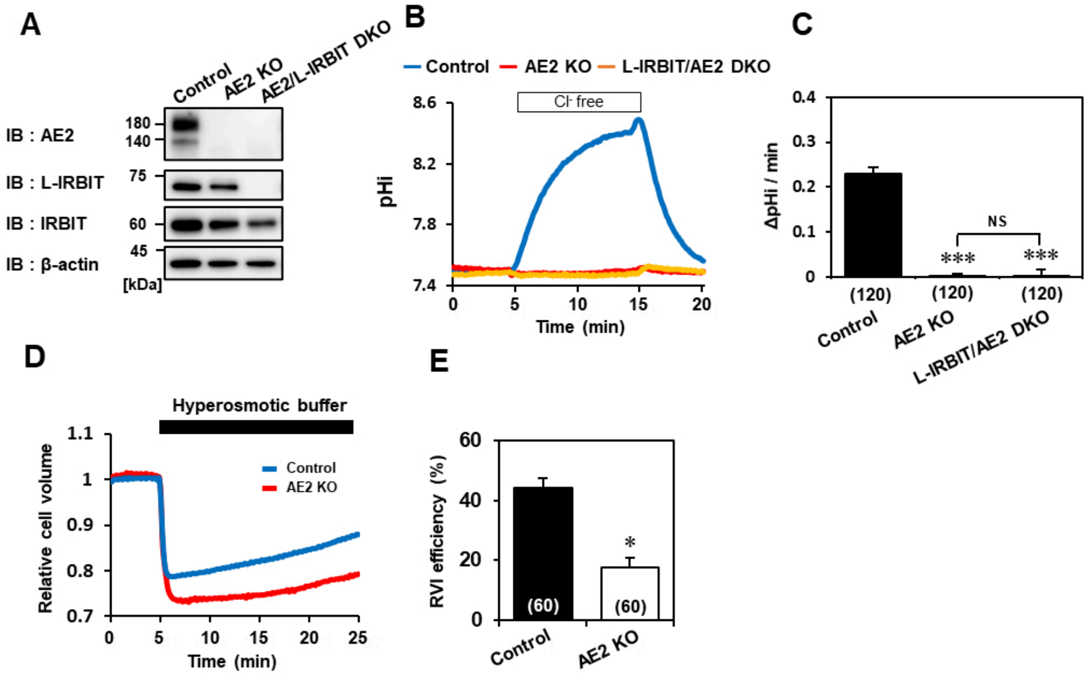

Figure S1. Cl^-/HCO_3^- anion exchange activity and cell volume recovery in AE2- or AE2/L-IRBIT double knockout cells. (A) AE2 and AE2/L-IRBIT double KO (DKO) cells were established by CRISPR/Cas9 strategy and their clones were verified for the expressions of IRBIT, Long-IRBIT and AE2 by immunoblot. (B) AE2 activity in AE2 KO cells or AE2/Long-IRBIT DKO cells was measured by intracellular pH change (pHi) upon changing perfusion buffer from Cl^--containing to Cl^--free ringer buffers with SNARF1 pH sensitive dye. A representative plot of pHi change obtained from control (bule), AE2 KO cells (red), and AE2/Long-IRBIT DKO cells (orange). (C) Average AE2 activity (ΔpHi/min) of each cells was 0.23 ± 0.02 (WT), 0.002 ± 0.003 (AE2 KO), and 0.001 ± 0.001 (L-IRBIT/AE2 DKO), N = 4. (D) Cell volume recovery in AE2 KO cells was measured by fluorescence change upon changing perfusion buffer from 300 mOsm buffer to 450 mOsm buffers with calcein-AM. A representative plot of fluorescence change as a relative cell volume from control (bule), AE2 KO clone1 (red). (E) RVI efficiency of each cells was 44.1 ± 3.3(WT), 17.6 ± 3.2 (AE2 KO), N = 3. The total cell numbers were indicated in each graph. The total cell numbers were indicated in each graph. $*P < 0.05$, $***P < 0.001$, NS (no significance).

Figure S2

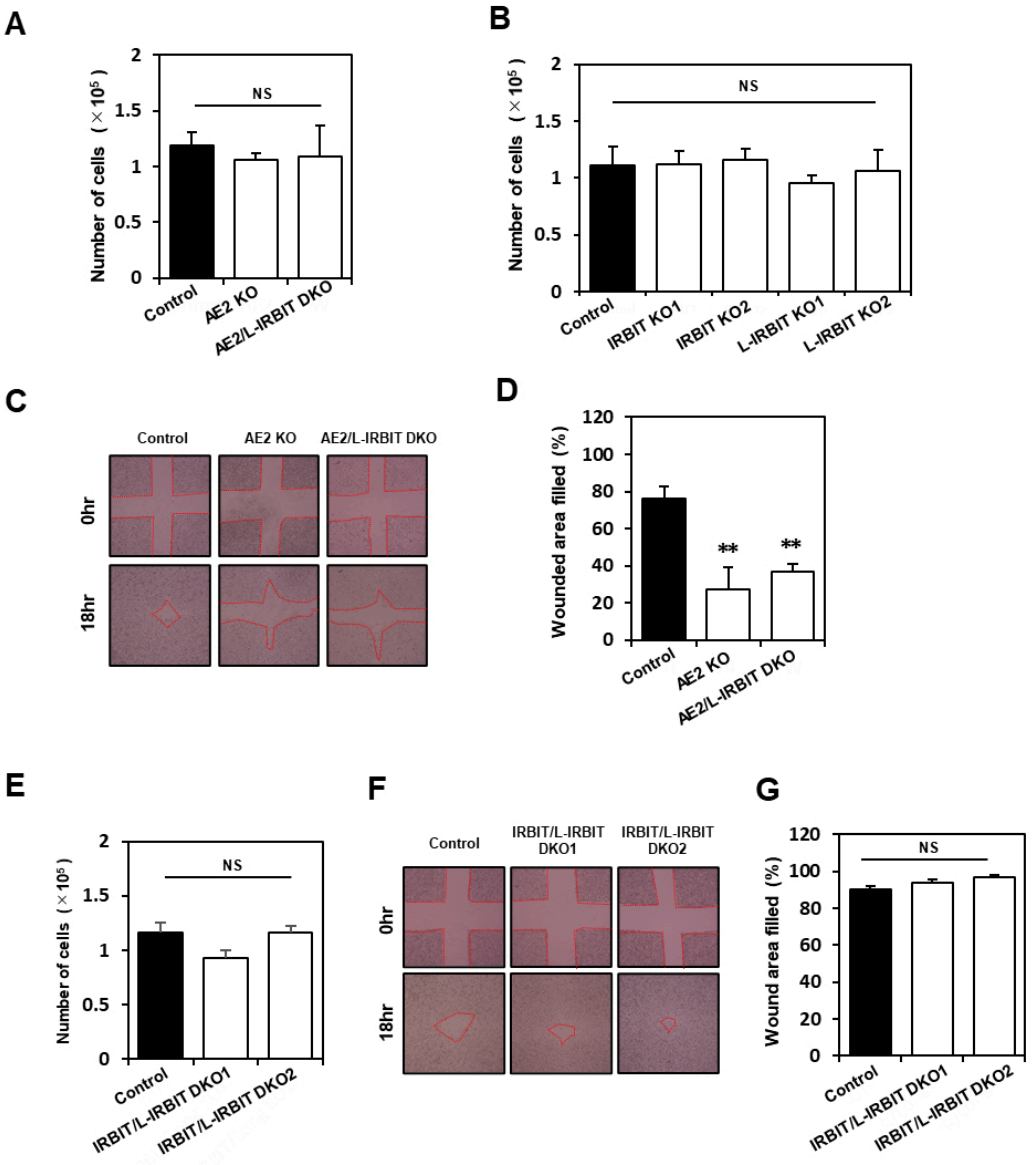

Figure S2. Cell growth and cell migration of IRBIT family- or AE2 knockout cells.

(A) The growth of WT , AE2 KO and AE2/L-IRBIT double knockout (DKO) cells determined by direct cell count using a hemocytometer at 24 h, N = 4. (B) The results of the wound healing assay were shown. Representative photomicrographs of the wounded cell monolayer are shown. (C) Wound width was measured in 6 positions immediately after wounding and 18 h later in WT, AE2 KO and AE2/Long-IRBIT DKO, N = 4. (D) The growth of in WT, IRBIT KO (IRBIT KO1, IRBIT KO2) and L-IRBIT KO (L-IRBIT KO1, L-IRBIT KO2) cells determined by direct cell count using a

hemocytometer at 24 h, N = 4. (E) The results of the wound healing assay were shown. Representative photomicrographs of the wounded cell monolayer are shown. (F) Wound width was measured in 6 positions immediately after wounding and 18 h later in WT, IRBIT KO (IRBIT KO1, IRBIT KO2) and L-IRBIT KO (L-IRBIT KO1, L-IRBIT KO2). N = 4. (G) The growth of in WT, IRBIT/L-IRBIT DKO (IRBIT/L-IRBIT DKO1, IRBIT/L-IRBIT DKO2) cells determined by direct cell count using a hemocytometer at 24 h, N = 4. (H) The results of the wound healing assay were shown. Representative photomicrographs of the wounded cell monolayer are shown. (I)Wound width was measured in 6 positions immediately after wounding and 18 h later in WT and IRBIT/L-IRBIT DKO (IRBIT/L-IRBIT DKO1, IRBIT/L-IRBIT DKO2), N = 4. *$P < 0.05$, **$P < 0.01$, NS (no significance).

Figure S3

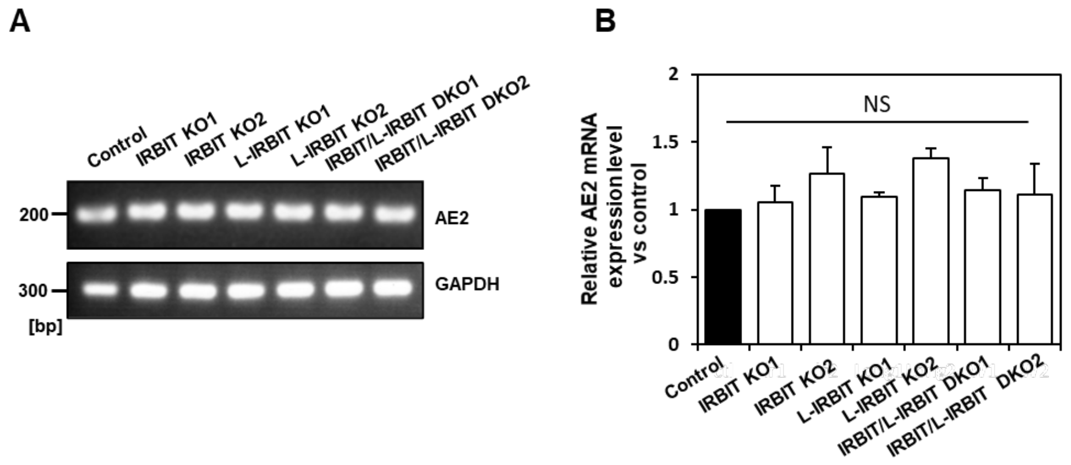

Figure S3. mRNA expression level of AE2 in IRBIT family knockout cells.

(A) mRNA expression level of AE2 and GAPDH were determined by RT-PCR using gene-specific primers. Representative data of amplified products by RT-PCR. (B) mRNA expression level of AE2 in each knockout cells was determined by qPCR using the delta Ct method ($2^{-\Delta\Delta Ct}$) with GAPDH as an internal control, N = 4. NS (no significance).

Figure S4

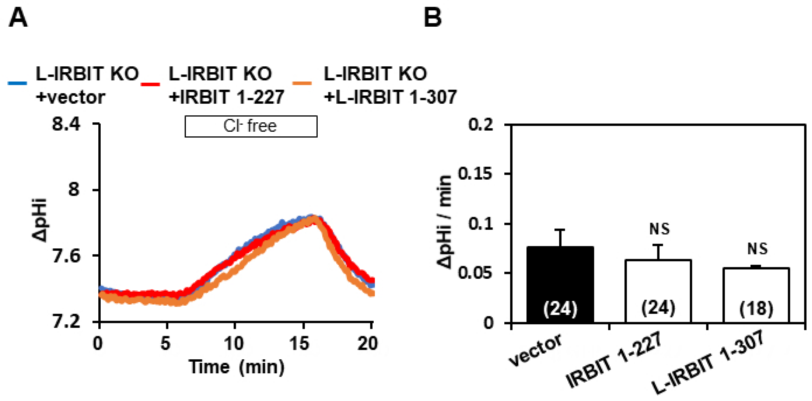

Figure S4. AE2 activity in L-IRBIT KO cells expressed with mutant IRBIT family proteins lacking the AE2 binding (A) The effects of the exogenous expression of IRBIT (aa 1-227) or L-IRBIT (aa 1-307) on AE2 activity in L-IRBIT KO cells were analyzed. IRBIT (aa 1-227) or L-IRBIT (aa 1-307) expressing cells were selected based on the GFP co-expressed signals. Blue trace is the L-IRBIT KO cells, a red trace is the L-IRBIT KO cells expressed with IRBIT (aa 1-227), an orange trace is the L-IRBIT KO cells expressed with L-IRBIT (aa 1-307). (B) Average AE2 activity in each cell type was 0.076 ± 0.017 (L-IRBIT KO + vector), 0.063 ± 0.015 (L-IRBIT KO + IRBIT (aa 1-227)), 0.054 ± 0.002 (L-IRBIT KO + L-IRBIT (aa 1-307), N = 3. The total cell numbers were indicated in each graph. NS (no significance).

Figure S5 to Figure S20 displayed unprocessed images of stained SDS-PAGE gels, Western blots and RT-PCR agarose gels used in the main figures and supplementary figure.

Figure S5. Full length silver-stained gel image of Figure 1A.

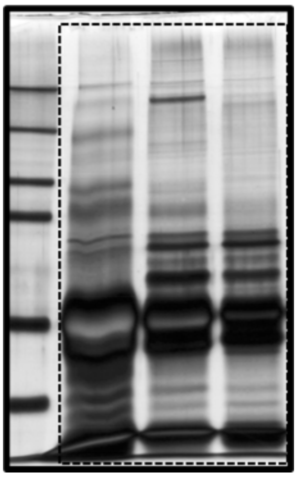

Figure S6. Full length blots of Figure 1B.

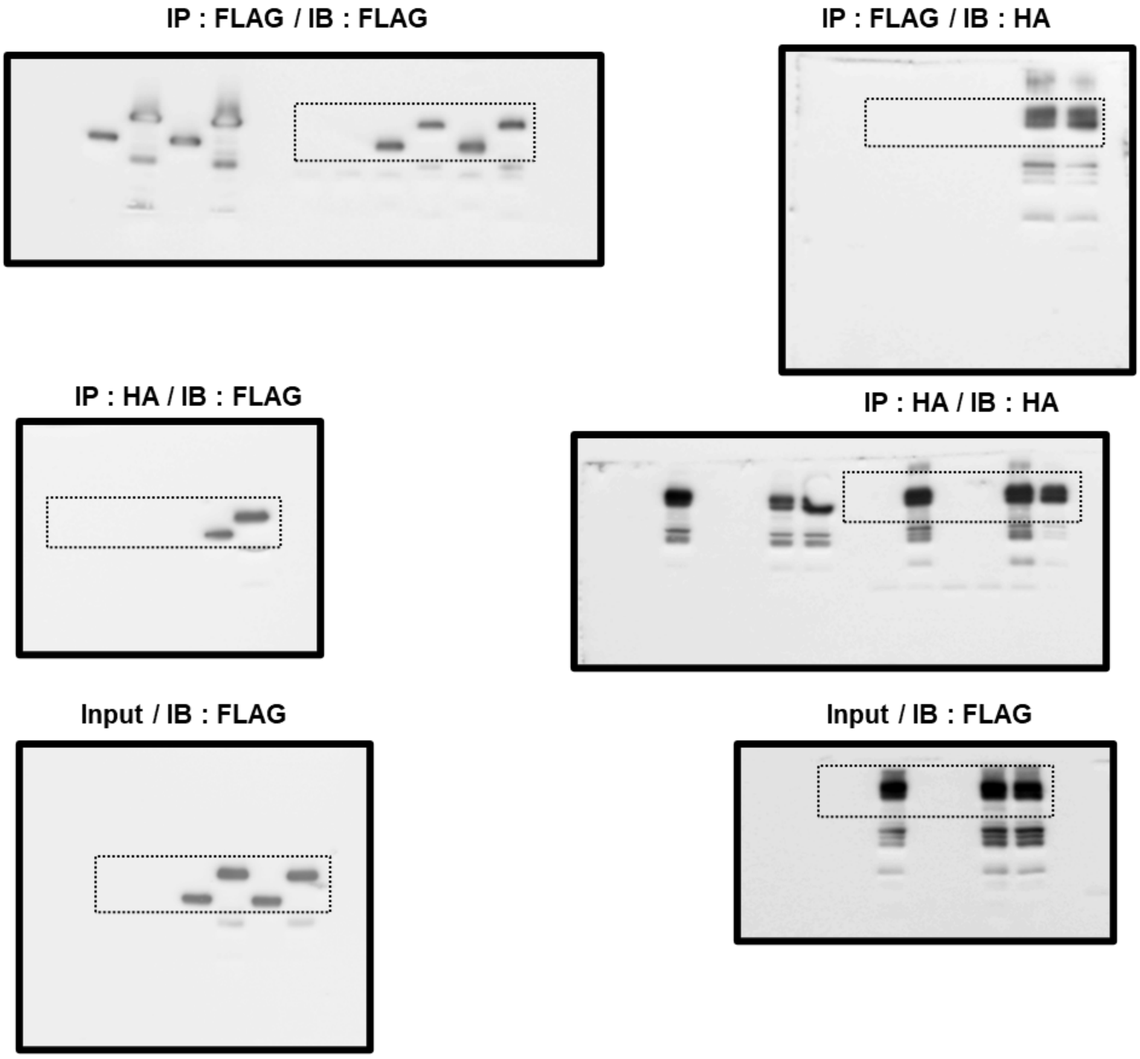

Figure S7. Full length blots of Figure 2A

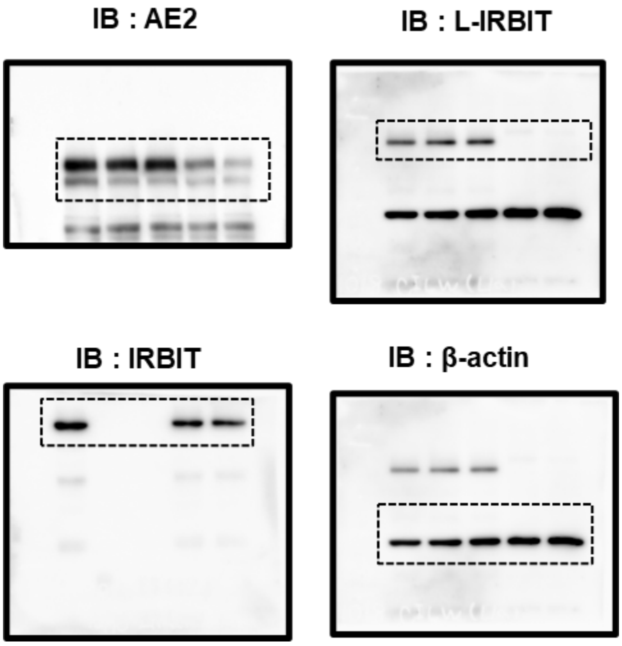

Figure S8-1. Full length blots of Figure 4B.

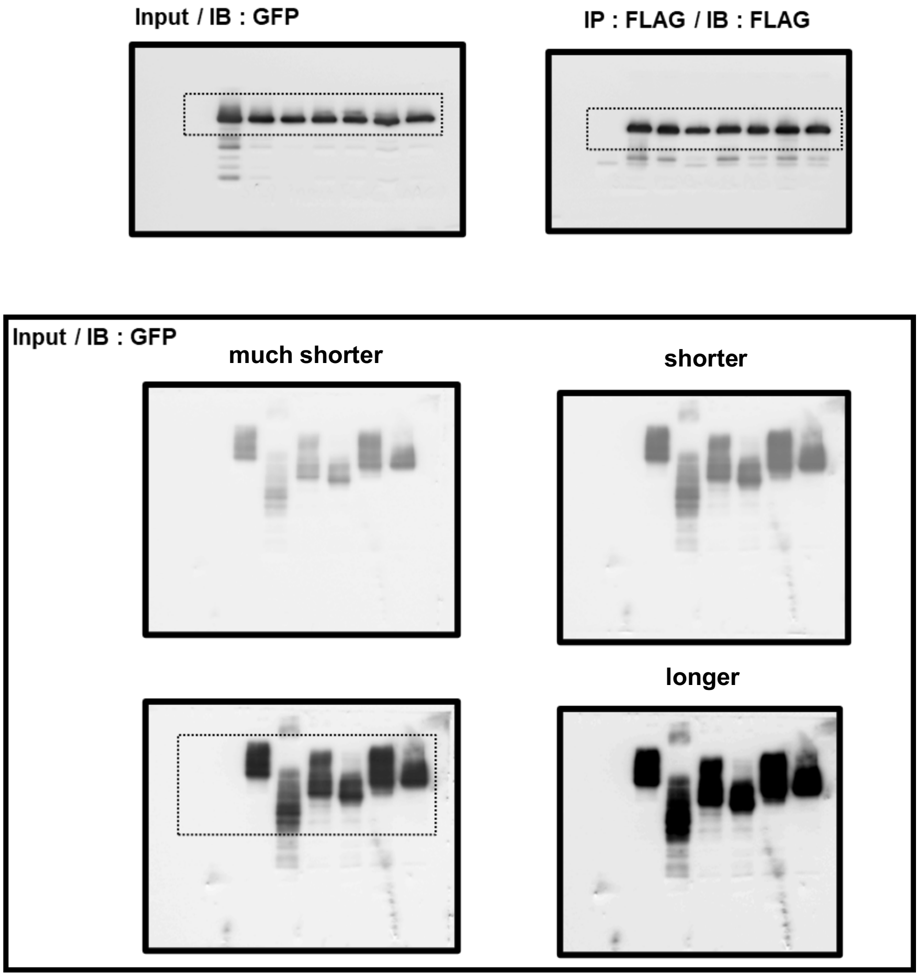

Figure S8-2. Full length blots of Figure 4B.

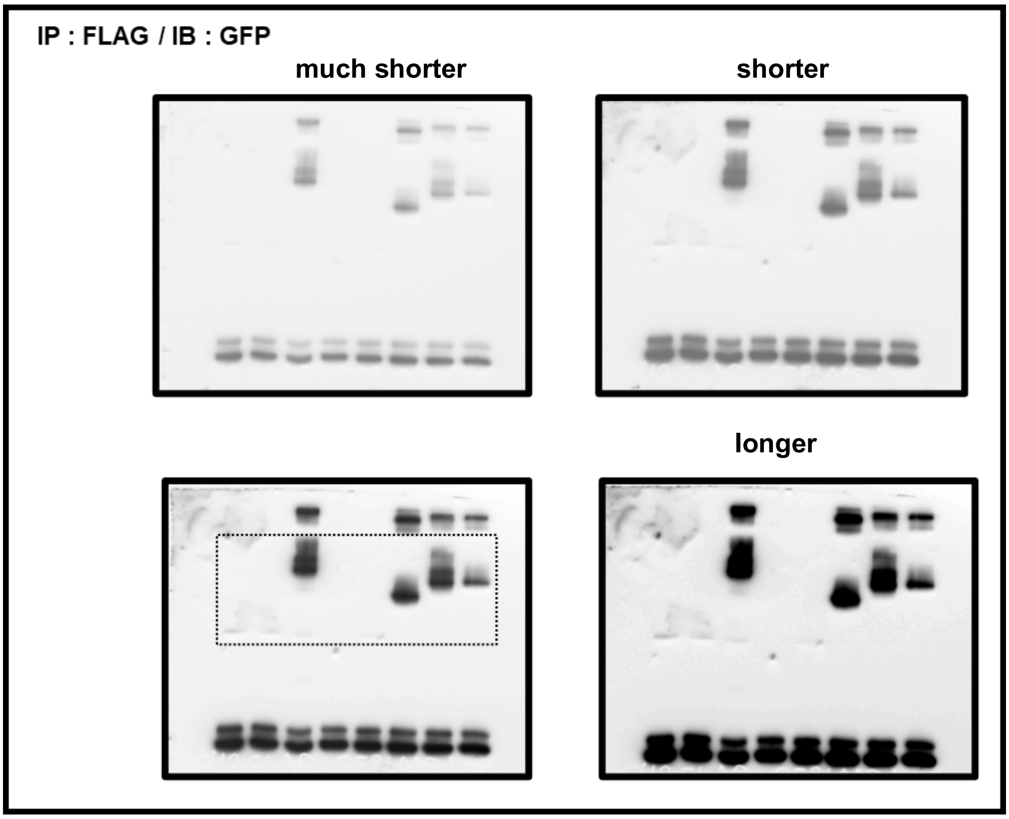

Figure S9. Full length blots and CBB-stained gels of Figure 4C.

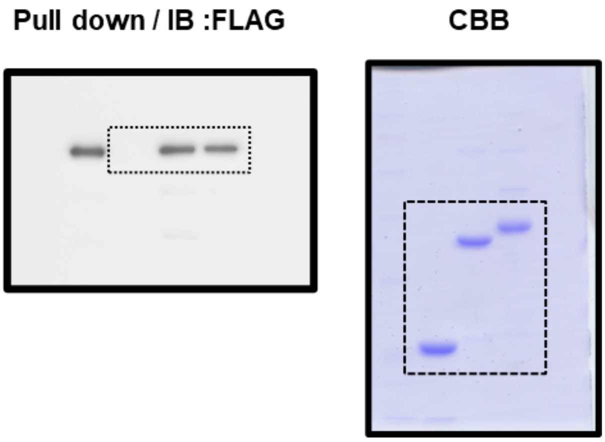

Figure S10. Full length blots and CBB-stained gels of Figure 4D.

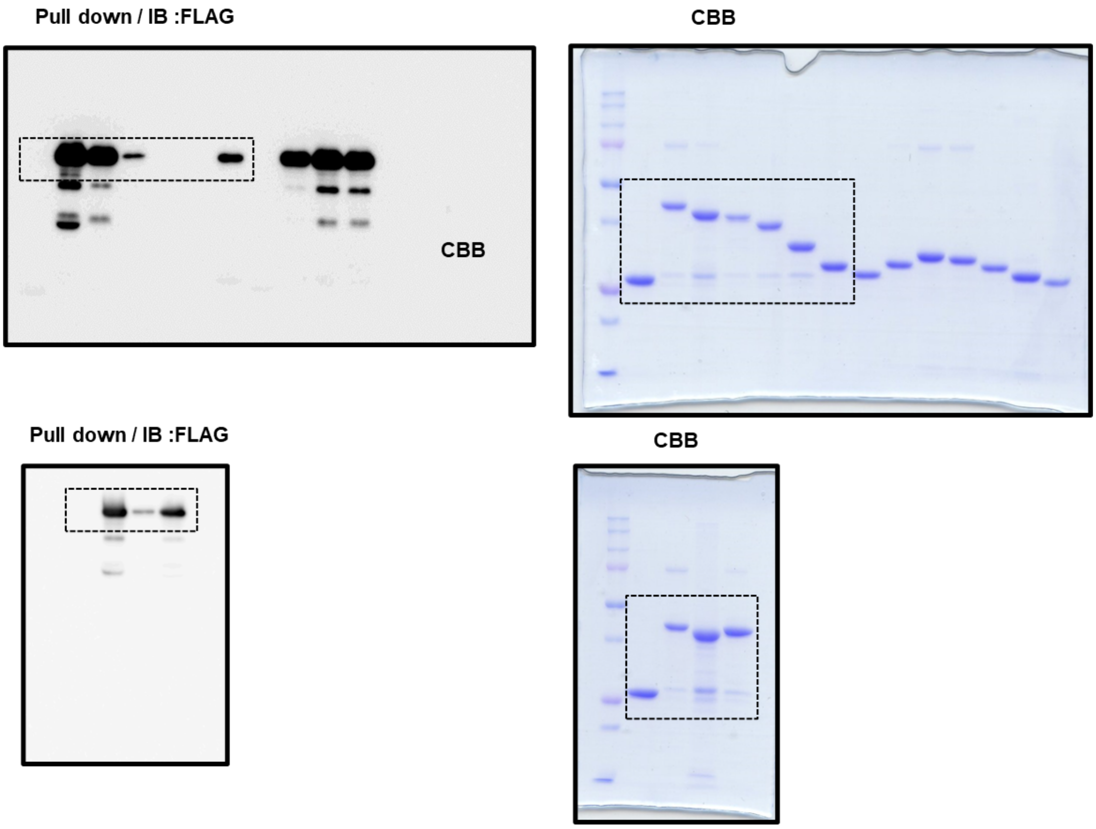

Figure S11. Full length blot of Figure 4E.

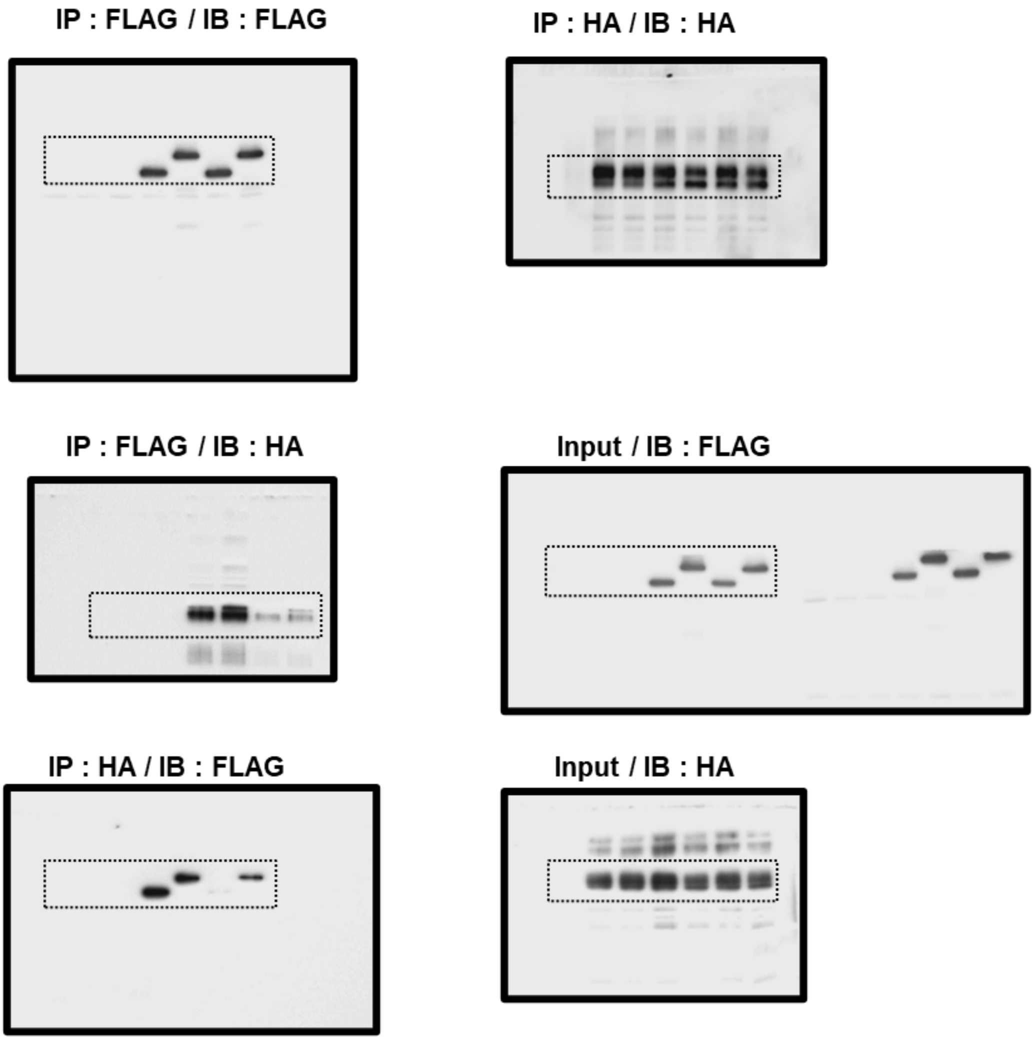

Figure S12. Full length blot of Figure 5B.

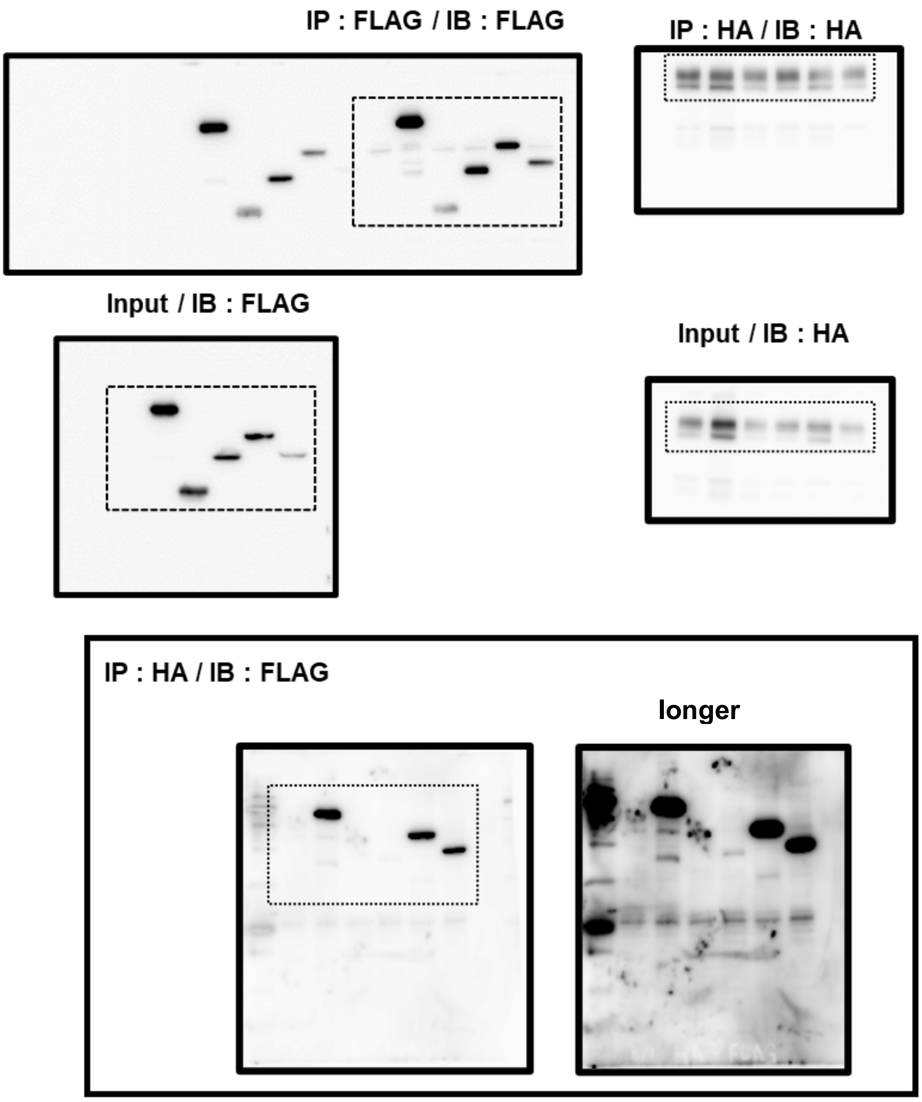

Figure S12-2. Full length blots of Figure 4B.

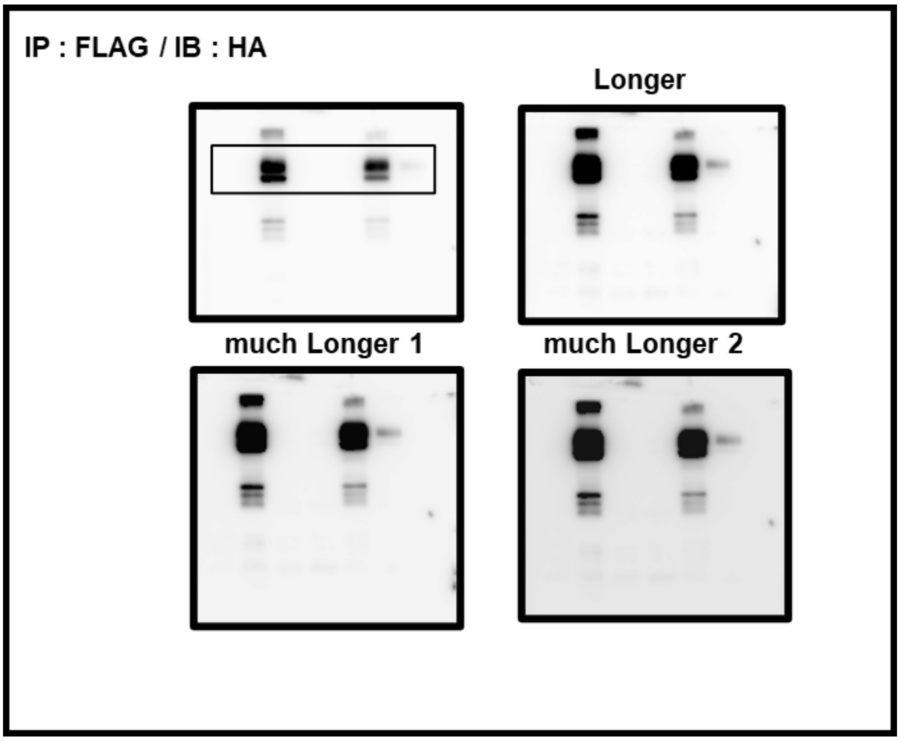

Figure S13. Full length blot and CBB-stained gel of Figure 5C.

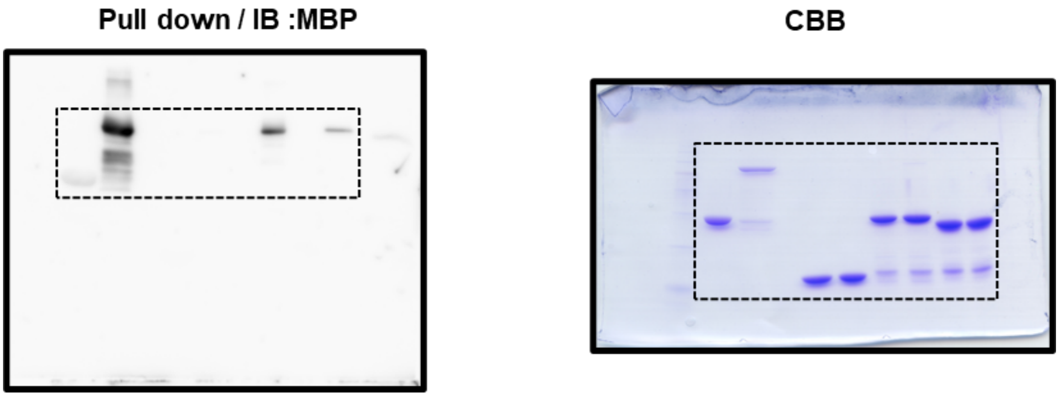

Figure S14. Full length blot of Figure 6B.

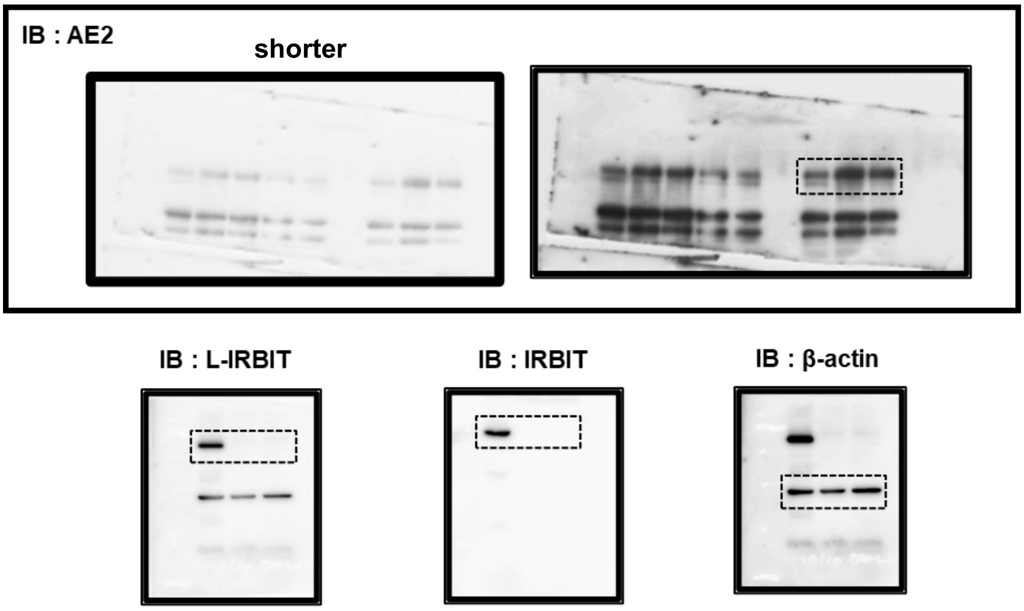

Figure S15. Full length blots of Figure 7A.

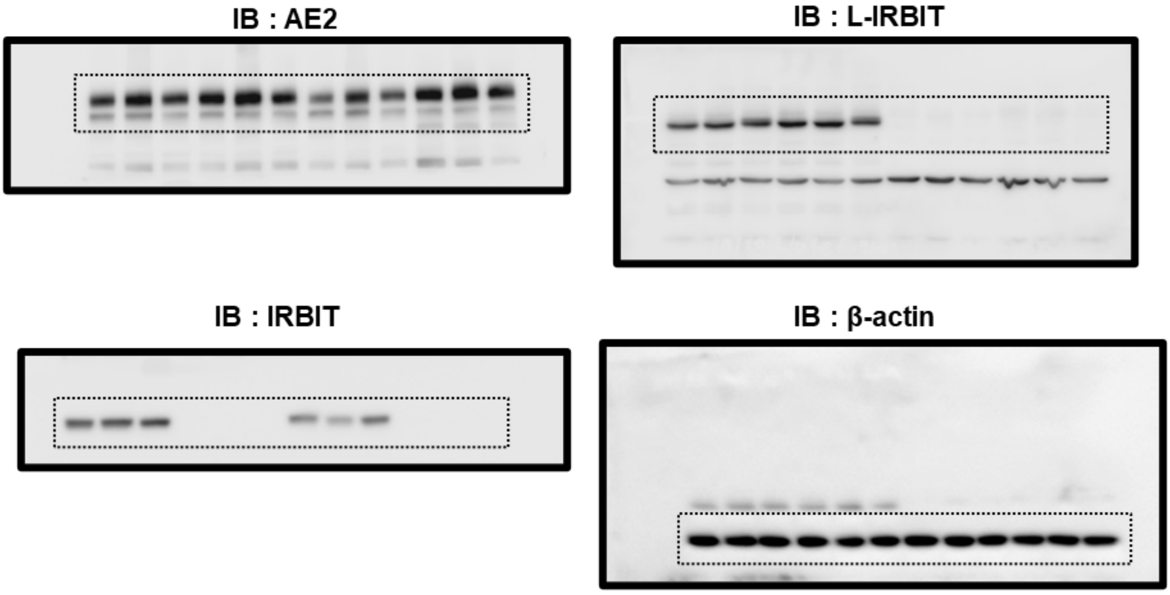

Figure S16. Full length blots of Figure 7B.

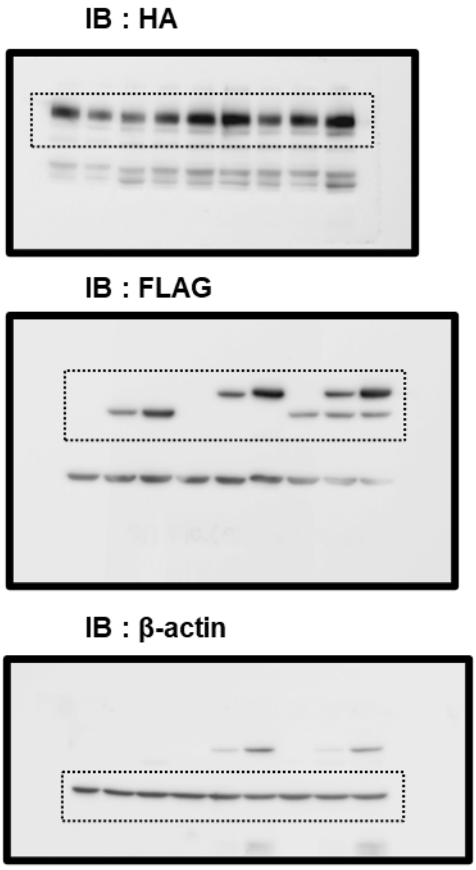

Figure S17. Full length blots of Figure 7C.

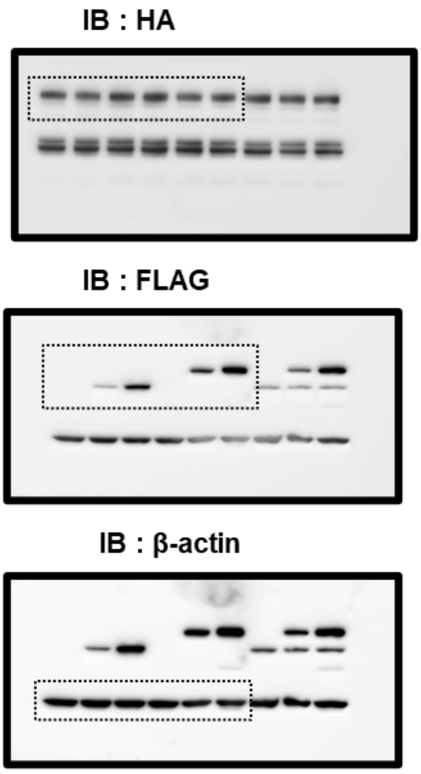

Figure S18. Full length blots of Figure 7D.

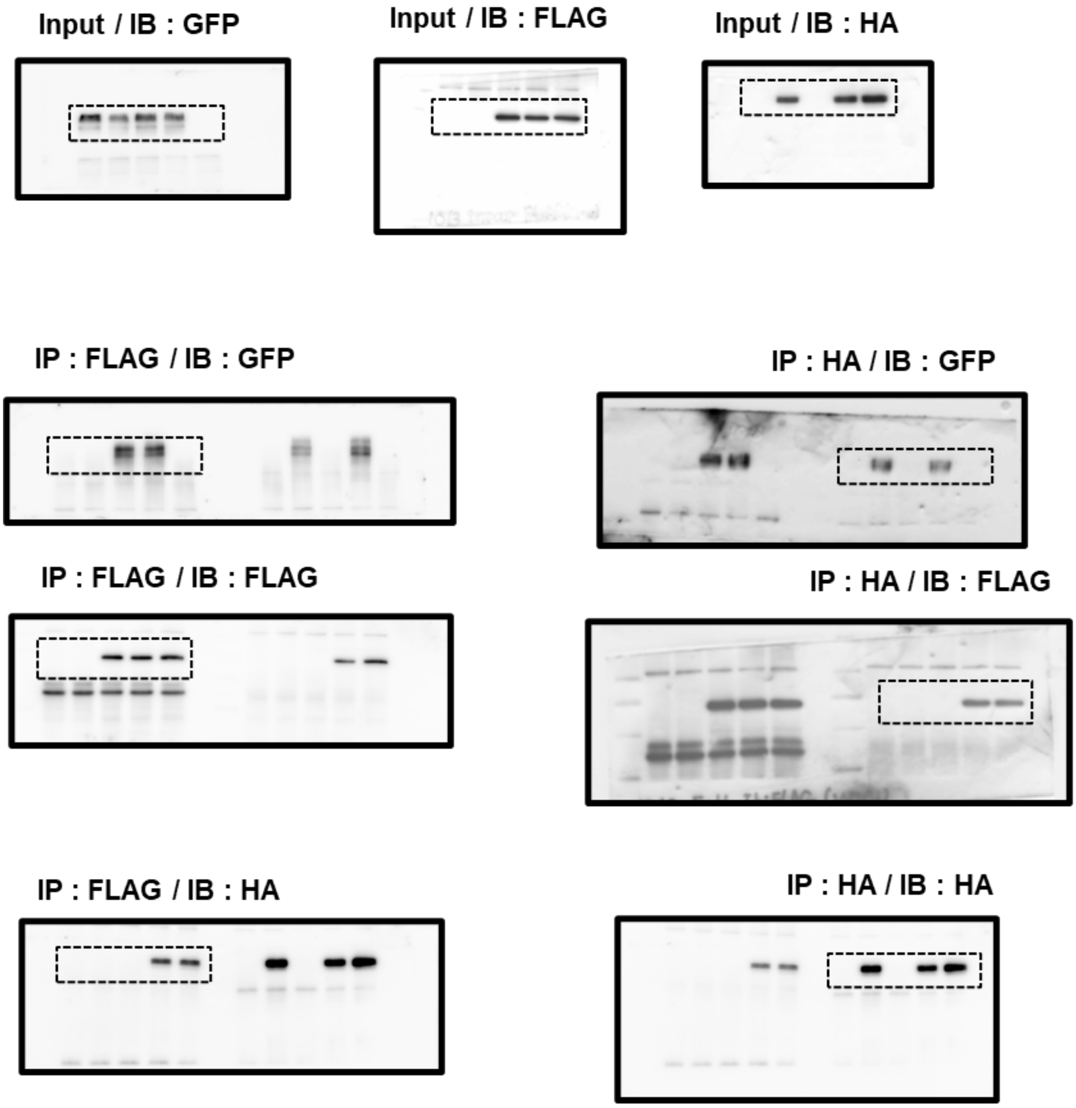

Figure S19. Full length blots of Figure S1A.

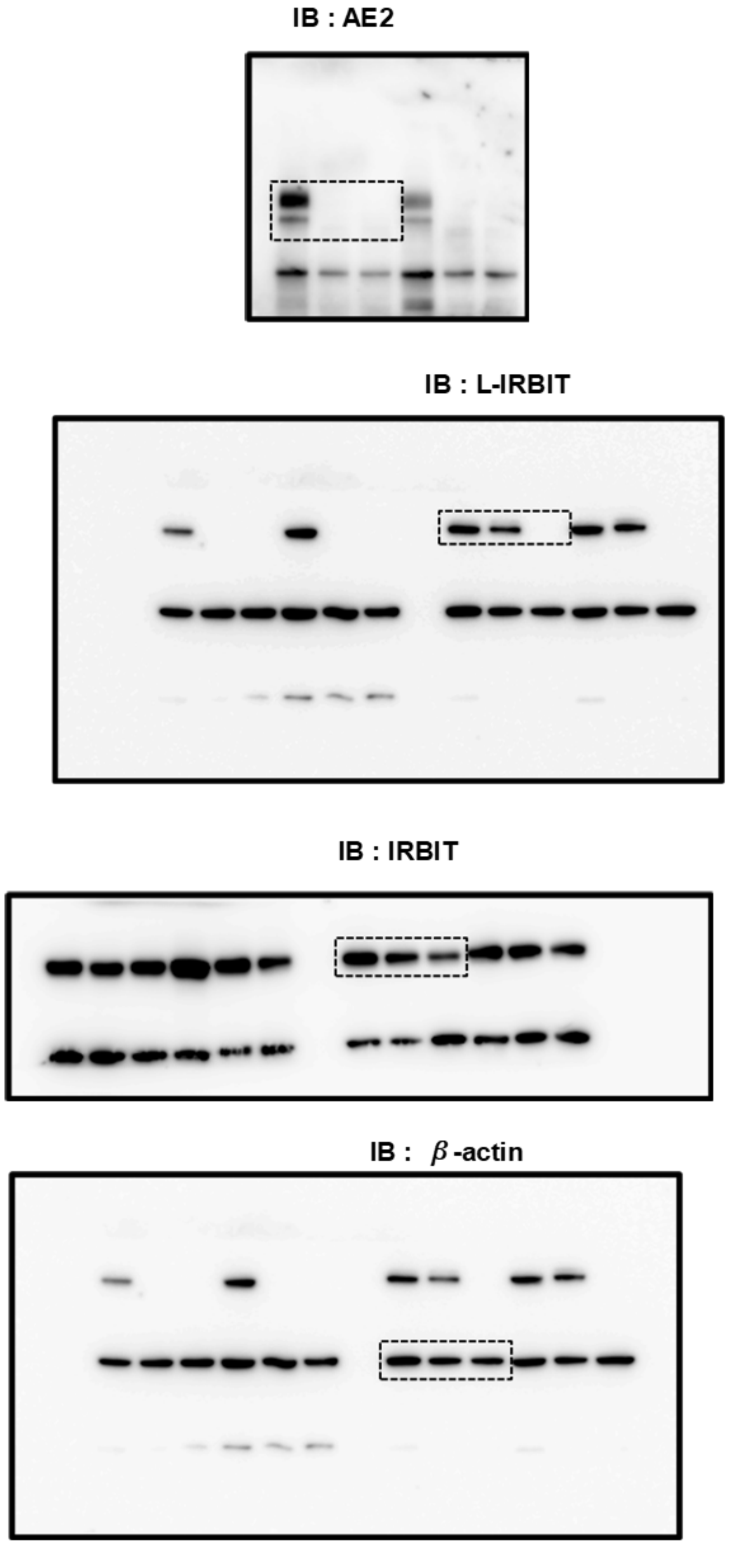

Figure S20. Full length agarose gels of Figure S3A.

AE2

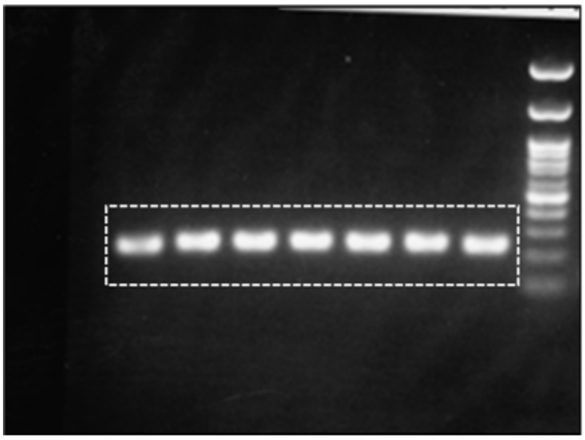

GAPDH

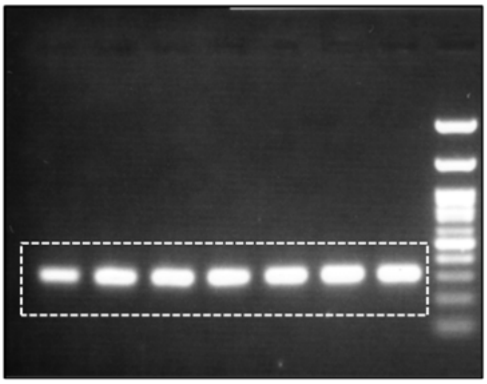

Supplement: Supplementary file 1 — Supplementary Information. [file 41598_2021_85499_MOESM1_ESM.pdf]
